# Breed‐Driven Microbiome Heterogeneity Regulates Intestinal Stem Cell Proliferation via *Lactobacillus*‐Lactate‐GPR81 Signaling

**DOI:** 10.1002/advs.202400058

**Published:** 2024-06-27

**Authors:** Haiqin Wu, Chunlong Mu, Xuan Li, Wenlu Fan, Le Shen, Weiyun Zhu

**Affiliations:** ^1^ Laboratory of Gastrointestinal Microbiology Jiangsu Key Laboratory of Gastrointestinal Nutrition and Animal Health College of Animal Science and Technology Nanjing Agricultural University Nanjing 210095 China; ^2^ National Center for International Research on Animal Gut Nutrition Nanjing Agricultural University Nanjing 210095 China; ^3^ Food Informatics AgResearch Te Ohu Rangahau Kai Palmerston North 4474 New Zealand; ^4^ Department of Surgery The University of Chicago Maryland Ave 60637 USA

**Keywords:** intestinal stem cell, lactobacillus, microbiome, small intestine, wnt/β‐catenin signaling

## Abstract

Genetically lean and obese individuals have distinct intestinal microbiota and function. However, the underlying mechanisms of the microbiome heterogeneity and its regulation on epithelial function such as intestinal stem cell (ISC) fate remain unclear. Employing pigs of genetically distinct breeds (obese Meishan and lean Yorkshire), this study reveals transcriptome‐wide variations in microbial ecology of the jejunum, characterized by enrichment of active *Lactobacillus* species, notably the predominant *Lactobacillus amylovorus* (*L. amylovorus*), and lactate metabolism network in obese breeds. The *L. amylovorus*‐dominant heterogeneity is paralleled with epithelial functionality difference as reflected by highly expressed GPR81, more proliferative ISCs and activated Wnt/β‐catenin signaling. Experiments using in‐house developed porcine jejunal organoids prove that live *L. amylovorus* and its metabolite lactate promote intestinal organoid growth. Mechanistically, *L. amylovorus* and lactate activate Wnt/β‐catenin signaling in a GPR81‐dependent manner to promote ISC‐mediated epithelial proliferation. However, heat‐killed *L. amylovorus* fail to cause these changes. These findings uncover a previously underrepresented role of *L. amylovorus* in regulating jejunal stem cells via *Lactobacillus*‐lactate‐GPR81 axis, a key mechanism bridging breed‐driven intestinal microbiome heterogeneity with ISC fate. Thus, results from this study provide new insights into the role of gut microbiome and stem cell interactions in maintaining intestinal homeostasis.

## Graphical abstract

Interactions between gut microbiome and stem cells in the jejunum, driven by genetic factors, are investigated in porcine breeds with genetically distinct phenotype. The jejunum of the obese breed harbors a *L. amylovorus*‐dominant microbiome, higher lactate, and more proliferative ISCs. Mechanistically, *L. amylovorus* regulates jejunal stem cell function via the *Lactobacillus*‐lactate‐GPR81 axis, linking breed‐driven microbiome heterogeneity with ISC fate.

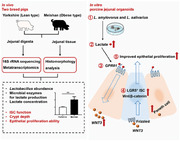

## 1 Introduction

It is well known that intestinal microbiota regulates intestinal homeostasis, including intestinal stem cell (ISC) proliferation and differentiation.^[^ ^1^ ^]^ This could be caused by microbiome control of several ISC niche signaling pathways including Wnt, Notch, Bone morphogenetic proteins (BMP) and Hedgehog that dictate the proliferation and differentiation fates of ISCs.^[^ ^2^ ^]^ Among the modulators of ISCs in crypt niches, the Wnt/β‐catenin pathway is the primary driving force for crypt formation and stem cell proliferation.^[^ ^3^ ^]^ In a murine model of *Citrobacter rodentium* infection, exogenous administration of *Lactobacillus reuteri* (*L. reuteri*) activated the Wnt/β‐catenin pathway and induced differentiation of stem cells toward Paneth cells to enhance mucosal barrier in the ileum.^[^ ^4^ ^]^ Administration of *Akkermansia muciniphila* BAA‐835 to mice could regulate the proliferation of ISCs and Wnt signaling,^[^ ^5^ ^]^ while *Bacillus subtilis* could protect mice against *Salmonella typhimurium* infection by promoting ISC differentiation through controlling Notch signaling in the ileum.^[^ ^6^ ^]^ The jejunum is the most important part of the small intestine for the digestion and absorption of food nutrients. However, signaling pathways that govern the ISC function and crypt organization, as well as their interaction with gut microbes in the jejunum, remain mysterious.

Different from the microbes observed in murine models, the jejunum of pigs harbors a different consortium of *Lactobacillus* predominant by *Lactobacillus amylovorus* (*L. amylovorus*).^[^ ^7^ ^]^ Other species such as *Limosilactobacillus mucosae* (*L. mucosae*) and *Ligilactobacillus salivarius* (*L. salivarius*) are also found in the jejunum.^[^ ^7^ ^]^ An increasing amount of evidence has documented that *Lactobacillus spp*. may exert different functionalities, for example, in regulating the intestinal barrier function.^[^ ^8^ ^]^ These findings raise the possibility that the strain‐level difference may lead to distinct impact on ISC function.

Genetic factors are well recognized as an inherent driven force shaping the phenotype and gut microbiome in mammals. Within a single mammal species, genetic variations contribute to distinct phenotypes of individuals.^[^ ^9^ ^]^ This is exemplified by the inter‐breed variation of gut microbiome and intestinal barrier function across pigs (Sus scrofa).^[^ ^10^, ^11^, ^12^ ^]^ By using a cross‐fostering model of Meishan and Yorkshire breeds, we previously showed that breed significantly impacts microbiome composition in colonic digesta and feces.^[^ ^13^, ^14^ ^]^ These findings suggest that phenotype variation observed in Meishan and Yorkshire porcine breeds may be related to intestinal homeostasis through controlling gut microbiota. Despite these findings, how breed‐driven intestinal microbiome heterogeneity contributes to the breed difference in intestinal epithelial function is less well understood.

Given their physiological similarity to humans^[^ ^15^ ^]^ and the presence of diverse *Lactobacillus* species,^[^ ^7^ ^]^ pigs with genetically distinct phenotypes offer a unique model for investigating the mechanism of microbiota‐ISC interactions at the jejunal interface. Considering the tight relationship between gut microbiome and epithelial function, here we hypothesize that breed‐driven intestinal microbiome heterogeneity contributes to the breed difference in ISC proliferation fate.

Employing obese‐type Meishan pigs and lean‐type Yorkshire pigs at the same physiological stage, the present study revealed significant inter‐breed differences. Specifically, we found higher *Lactobacillus* abundances and lactate‐producing enzyme‐encoding genes in the jejunum of Meishan than Yorkshire pigs, which corresponded with increased proliferating crypt cells and higher Wnt/β‐catenin signaling. Furthermore, using an in‐house developed porcine organoid model, we demonstrated that *L. amylovorus*, one of the most dominant *Lactobacillus* species in the porcine jejunum,^[^ ^7^ ^]^ promoted ISCs‐mediated proliferation through activating Wnt/β‐catenin signaling in a lactate‐GPR81 dependent manner. These findings underscore the physiological relevance of breed‐specific microbiome variance in regulating ISC activity in the jejunum, highlighting a novel mechanism by which the gut microbiome influences epithelial function.

## 2 Results

### 2.1 Meishan Pigs Have Higher Jejunal *Lactobacillus* and Lactate Than Yorkshire Pigs

**Figure 1:**
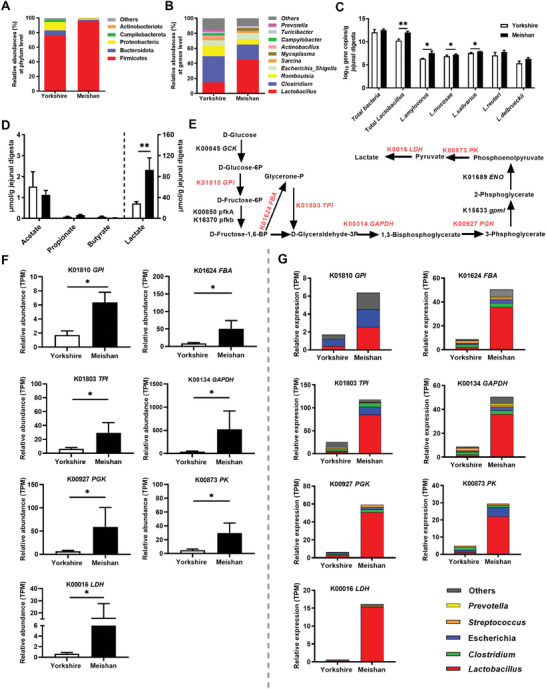
Microbial composition, metabolites and metabolic pathways for microbial lactate production in the jejunum in Meishan and Yorkshire pigs. A) Relative abundance of bacteria at phylum level in the jejunal digesta (n = 6). B) The relative abundance of microbial genus‐level in the jejunal digesta (n = 6). C) Quantitative real‐time PCR measurement of dominant *Lactobacillus* quantities in the jejunal digesta. (Graphs represent mean ± SEM, n = 6). D) SCFAs and lactate concentrations in jejunal digesta (Graphs represent mean ± SEM, n = 6). E) Schematic presentation of metabolic pathways for microbial lactate production from carbohydrates. Significantly different genes encoding enzymes involved in lactate production are shown in red (increased in the Meishan group). F) Expression of genes involved in lactate production (Graphs represent mean ± SEM, n = 4). G) Phylogenetic distribution of sequences in lactate producing genes assigned to the identified genus. Only genera with functional gene abundance of more than 1 TPM in least one group are presented (n = 4). Mann‐Whitney *U* test was performed between two groups while asterisks mean statistically significant difference: *P ≤ 0.05, **P ≤ 0.01.

To explore whether phenotype variation in a single species can affect gut microbial composition, we compared the jejunal microbiota of Meishan (obese type) pig and Yorkshire (lean type) pig by 16S rRNA gene sequencing. Overall microbiota communities, as revealed by α diversity and β diversity (Figure S1A,B, Supporting Information), were not significantly different between the two breeds. At the phylum level, Firmicutes was the dominant phylum (Table S1, Supporting Information) in the jejunal digesta in both breeds (**Figure** **1**A), with higher abundance in Meishan pigs than Yorkshire pigs (*P* = 0.030, Figure S1C, Supporting Information). At the genus level, *Lactobacillus* was the dominant genus (Figure 1B and Table S2, Supporting Information), with higher relative abundance in Meishan pigs than Yorkshire pigs (*P* = 0.041, Figure S1D, Supporting Information). Quantitative copy number analysis also demonstrated that while total bacterial loads were similar between these two breeds, Meishan breed had more abundant *Lactobacillus* than the Yorkshire breed in jejunal digesta (*P* = 0.0043). At the species level, *L. amylovorus* (*P* = 0.041), *L. mucosae* (*P* = 0.033) and *L. salivarius* (*P* = 0.024) were more abundant in Meishan pigs than Yorkshire pigs, while no differences were observed for *L. reuteri* (*P* = 0.373) and *Lactobacillus delbrueckii* (*L. delbrueckii*) (*P* = 0.094) between two breeds (Figure 1C).

Short‐chain fatty acids (SCFAs) and lactate are some of the major microbial products in the intestinal contents. In jejunal digesta, both breeds had similar SCFAs concentrations (acetate, propionate, and butyrate), whereas lactate concentration was higher in Meishan pigs (*P* = 0.008, Figure 1D). These results indicate that the Meishan pigs have higher jejunal *Lactobacillus* and lactate content than Yorkshire pigs.

### 2.2 The Jejunal Microbiota of Meishan Pigs Expresses Higher Levels of Enzymes for Lactate Production Than Yorkshire Pigs

It is well known that *Lactobacillus* contributes to lactate production. To test if lactate in the jejunum of Meishan pigs is caused by *Lactobacillus* activity, shotgun metatranscriptome sequencing was performed on jejunal microbiome. By mapping the KEGG orthologous (KO) genes involved in carbohydrate metabolism, the abundance of functional genes involved in lactate production was determined (Figure 1E,F). Within the glycolysis pathway, seven enzymes, including glucose‐6‐phosphate isomerase (K01810, *GPI*, *P* = 0.029), fructose bisphosphate aldolase (K01624, *FBA*, *P* = 0.029), triosephosphate isomerase (K01803, *TPI*, *P* = 0.029), glyceraldehyde 3‐phosphate dehydrogenase (K00134, *GAPDH*, *P* = 0.029), phosphoglycerate kinase (K00927, *PGK*, *P* = 0.029), pyruvate kinase (K00873, *PK*, *P* = 0.029) and L‐lactate dehydrogenase (K0016, *LDH*, *P* = 0.029), had higher RNA abundances in Meishan pigs than Yorkshire pigs (Figure 1E,F). In addition, the majority of genes encoding the seven enzymes identified above were phylogenetically assigned to the genus *Lactobacillus* (Figure 1G). These results indicate that *Lactobacillus‐*produced enzymes for glycolysis contribute to higher lactate concentration in the jejunal digesta of Meishan pigs relative to Yorkshire pigs.

### 2.3 Meishan Pigs Have an Enhanced Crypt Stem Cell Function and Pro‐Proliferative Signaling in the Jejunum Relative to Yorkshire Pigs

**Figure 2:**
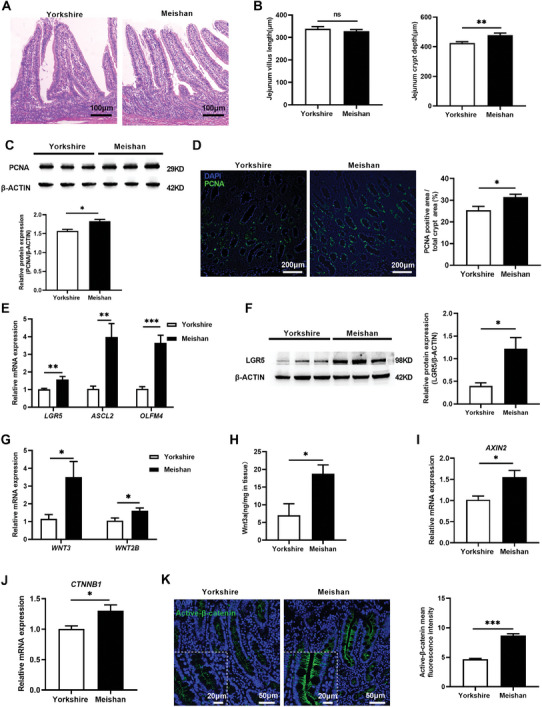
Histology, proliferative cells and aISC expression, Wnt/β‐catenin signaling in the jejunum of Meishan and Yorkshire pigs. A) Representative histological micrographs. Scale bar = 100 µm. B) Quantitative analysis of jejunal villus height and crypt depth (n = 6). C) Western blot analysis of PCNA protein expression in jejunum samples (n = 3). Up panel: representative blot. Down panel: data quantification. D) Immunofluorescent imaging of PCNA stained jejunal sections (n = 6). Left: representative images (green: PCNA, blue: DNA, scale bar = 200 µm). Right: Quantification percentage of PCNA positive cells. E) Relative mRNA expression of aISC marker genes: *LGR5*, *ASCL2* and *OLFM4* (n = 6). F) Western blot analysis of LGR5 protein expression in jejunal samples (n = 3). Left panel: representative blot. Right panel: Quantification of LGR5 expression. G) Jejuna*l WNT3* and *WNT2B* mRNA expression (n = 6). H) Quantification of WNT3A protein abundance in jejunal tissue homogenates from the homogenized jejunum (n = 6). I) Relative expression of *AXIN2* mRNA (n = 6). J) Relative expression of *CTNNB1* mRNA (n = 6). K) Immunofluorescent imaging of active β‐catenin stained jejunal sections (n = 6). Left panel: representative images (green: active β‐catenin, blue: DNA). Right panel: Quantification of β‐catenin mean fluorescence intensity. Graphs represent mean ± SEM. The Student's *t*‐test was performed between two groups while asterisks mean statistically significant difference: *P ≤ 0.05, **P ≤ 0.01, ***P ≤ 0.001, *P* > 0.05 no significance (ns).

Gut microbiota and metabolites play a critical role in regulating intestinal physiology.^[^ ^16^, ^17^, ^18^ ^]^ Our earlier studies and present results indicate that Meishan and Yorkshire breeds have phenotype‐dependent differences in microbiome and intestinal immune function.^[^ ^13^, ^14^ ^]^ This suggests a link between phenotype‐dependent microbiome and gut function. To address this possibility, we first determined jejunal tissue organization and signaling differences between Meishan and Yorkshire breeds. Histological studies showed that jejunal villus height was similar in both breeds, while Meishan pigs had significantly deeper crypts than Yorkshire pigs (*P* = 0.002, **Figure** **2**A and B). RT‐qPCR and western blot for proliferating cell nuclear antigen (PCNA), a proliferation marker, showed that Meishan pigs has higher PCNA protein abundance than Yorkshire pigs (*P* = 0.042, Figure 2C). Immunofluorescent staining of jejunal tissue sections further showed that within the jejunal crypt, fraction of PCNA‐positive area relative to total crypt area was higher in the Meishan breed (*P* = 0.015, Figure 2D). These results show that Meishan pigs had higher jejunal epithelial proliferation relative to Yorkshire pigs.

With in the intestinal crypt, epithelial proliferation can be impacted by ISC function. We next tested if altered ISC function could contribute to increased jejunal epithelial proliferation in Meishan pigs. The rapid proliferating active ISC (aISC) is the major ISC subtype that is responsible for intestinal epithelial replenishment at homeostatic state. Thus, we determined the properties of aISCs, including markers such as LGR5, ASCL2, and OLFM4,^[^ ^19^, ^20^ ^]^ in the jejunal tissue of both breeds of pig. RT‐qPCR of jejunal segments showed that Meishan pigs had higher expression of aISC markers *LGR5* (*P* = 0.007), *ASCL2* (*P* = 0.004), and *OLFM4* (*P* = 0.0002) (Figure 2E) and western blot of jejunal segment lysates showed LGR5 protein abundance was also increased in Meishan pigs (*P* = 0.031, Figure 2F). Overall, these results demonstrate that Meishan pigs have higher aISC function, which may explain the increased crypt proliferation.

Wnt/β‐catenin signaling pathway is a major driver for ISC proliferation,^[^ ^3^, ^21^ ^]^ and both *LGR5* and *ASCL2* are Wnt target genes.^[^ ^22^, ^23^, ^24^ ^]^ Thus we speculated that intestinal proliferation and stem cell marker differences between the two breeds are caused by distinct Wnt/β‐catenin signaling strength. To test this, we first measured Wnt ligand expression in jejunal tissue segments. Meishan pigs had higher *WNT3* (*P* = 0.028) and *WNT2B* (*P* = 0.025) mRNA expression relative to Yorkshire pigs (Figure 2G), and protein quantification showed that WNT3A protein was more abundant in jejunal tissue lysates from Meishan pigs (*P* = 0.015, Figure 2H). We subsequently determined Wnt signaling activity in jejunal tissue segments. RT‐qPCR studies showed that Meishan pigs had higher *AXIN2* (a Wnt target gene) mRNA expression (*P* = 0.014, Figure 2I). Furthermore, RT‐qPCR showed that *CTNNB1* (β‐catenin) mRNA was higher in Meishan jejunal segments (*P* = 0.0078, Figure 2J), and immunofluorescent staining showed increased active‐β‐catenin fluorescence intensity and higher accumulation of active β‐catenin in the nucleus of jejunal crypt in Meishan pigs (*P* < 0.0001, Figure 2K), demonstrating increased β‐catenin activity. Taken together, these results show that the jejunal tissue of Meishan pigs has higher Wnt/β‐catenin signaling activity.

It is reported that lysozyme‐producing Paneth cells are a driver for crypt proliferation, at least partially through Wnt ligand production. Thus, we determined Paneth cell abundance in jejunal crypts. RT‐qPCR and western blot analyses showed that Meishan breed had higher lysozyme gene expression (*P* = 0.002) and protein expression (*P* = 0.041), respectively (Figure S2A and B, Supporting Information). Immunohistochemical staining showed increased Paneth cell number (*P* < 0.001, Figure S2C, Supporting Information), which was also supported by UEA‐1 staining (Figure S2D, Supporting Information). The increased Paneth cell abundance in Meishan pigs may drive Wnt signaling to impact ISC function.

### 2.4 Dominant *Lactobacillus* Species *L. amylovorus* and *L. salivarius* Promote Porcine Jejunal Organoid Proliferation Through Activating Wnt/β‐catenin Signaling

To mechanistically explore the role of intestinal microbes on ISC‐mediated porcine epithelial proliferation, we established jejunal intestinal organoids from Yorkshire breed (Figure S3A, Supporting Information).^[^ ^25^ ^]^ In addition to progressive increases in organoid size and bud following passaging (Figure S3B–D, Supporting Information), abundances of markers for aISCs (*LGR5*), +4 quiescent stem cells (*BMI1*), enterocytes (*FABP* and *VILLIN*), proliferative cells (*PCNA*), Paneth cells (*LYZ1*), enteroendocrine cells (*CHGA*) and goblet cells (*MUC2*) were all upregulated over time (Figure S3E–H, Supporting Information). These data demonstrate that this organoid model is suitable for mechanistic investigations of how *Lactobacillus* and its metabolic product may impact jejunal epithelial behavior.

Our previous studies reported that *L. amylovorus* was one of the most dominant species within *Lactobacillus* in the small intestine of pigs,^[^ ^7^ ^]^ and our results above showed *L. amylovorus* (Figure 1G) was significantly higher in the jejunal digesta of Meishan pig. Therefore, we tested how *L. amylovorus* may impact intestinal epithelial growth and signaling. We utilized the *L. amylovorus* S1 strain, a strain we previously isolated from jejunal digesta of pigs,^[^ ^7^ ^]^ to treat the porcine jejunal epithelial organoids. To determine dose dependency of *L. amylovorus* S1 treatment, different doses including 10^3^, 10^4^, 10^5^, 10^6^ and 10^7^ CFU of live *L. amylovorus* were used to treat porcine jejunal organoids for 48 h. Treatments with 10^3^ and 10^4^ CFU of *L. amylovorus* had no impact on the growth of intestinal organoids (Figure S4A–D, Supporting Information). Treatment with 10^5^ CFU of *L. amylovorus* significantly promoted intestinal organoid proliferation (Figure S4A–D, Supporting Information). The dose of 10^6^ CFU of *L. amylovorus* further increased intestinal organoid proliferation (Figure S4A–D, Supporting Information) relative to mock‐treated organoids, with increasing mRNA abundance of proliferation marker *PCNA* (*P* = 0.0004), aISC markers including *LGR5* (*P* < 0.0001), *ASCL2* (*P* < 0.0001) and *OLFM4* (*P* < 0.0001), and Wnt/β‐catenin signaling related genes *WNT3* (*P* < 0.0001), *AXIN2* (*P* = 0.0022), and *CTNNB1* (*P* = 0.0049) (Figure S4E–I, Supporting Information). At the higher dose of 10^7^ CFU, *L. amylovorus* had no impact on the growth of intestinal organoids (Figure S4A–D, Supporting Information), but decreased the mRNA expressions of *PCNA* (*P* = 0.0202), *LGR5* (*P* = 0.0316), *ASCL2* (*P* = 0.0257), and *OLFM4* (*P* = 0.0183) (Figure S4E–H, Supporting Information). Taken together, these results indicate that the dose of 10^6^ CFU of *L. amylovorus* is the most appropriate dosage to test the effect of *L. amylovorus* on organoid physiology.

In addition to *L. amylovorus*, we also tested the impact of *L. mucosae* and *L. salivarius*, whose relative abundance was also higher in the Meishan breed, on jejunal organoid proliferation and related signaling. While *L. salivarius* was also a strong driver for jejunal organoid growth and Wnt signaling (Figure S5A–G, Supporting Information), *L. mucosae* only had a modest effect on organoid growth and Wnt signaling (Figure S5A–G, Supporting Information).

**Figure 3:**
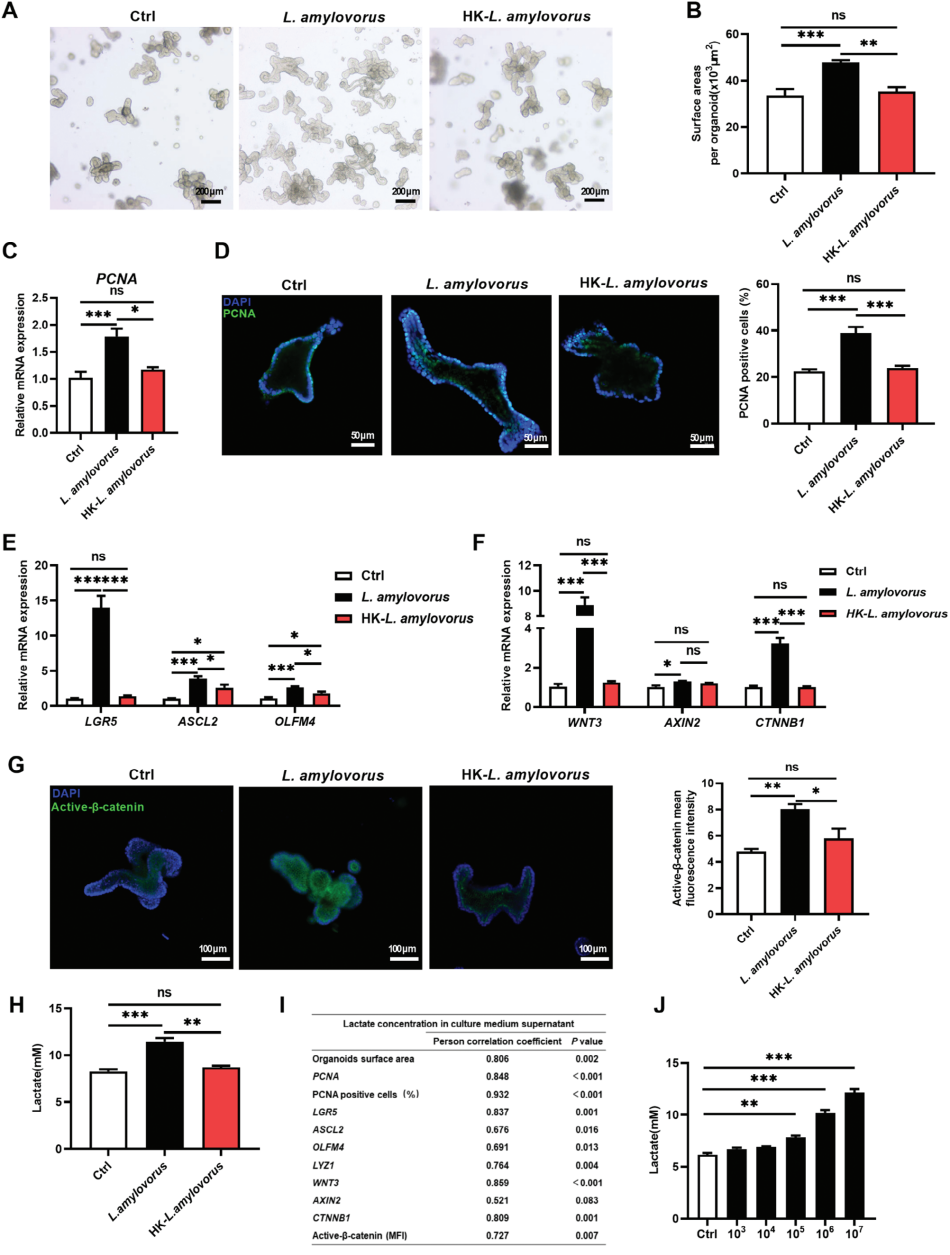
Effect of *L. amylovorus* and HK‐*L. amylovorus* on porcine jejunal organoid proliferation and Wnt/β‐catenin signaling. A) 10^6^ CFU *L. amylovorus and* HK*‐L. amylovorus* was used respectively, to treat organoids for 48 h. Organoids morphology was assessed by light microscopy (Scale bar = 200 µm). B) Quantification of organoid area (n = 4 wells per group), 15 organoids per well). C) Relative mRNA expression of *PCNA* (n = 4 wells per group). D) Immunofluorescent imaging of PCNA stained porcine jejunal organoids (n = 4 wells per group). Left: representative images (green: PCNA, blue: DNA, scale bar = 50 µm). Right: Quantification percentage of PCNA positive cells (15 organoids per well). E) Relative mRNA expression of *LGR5*, *ASCL2* and *OLFM4* in jejunal organoids (n = 4 wells per group). F) Relative mRNA expression of Wnt/β‐catenin signaling related genes *WNT3*, *AXIN2* and *CTNNB1* in organoids (n = 4 wells per group). G) Immunofluorescent imaging of Active‐β‐catenin stained porcine jejunal organoids (n = 4 wells per group). Left: representative images (green: Active‐β‐catenin, blue: DNA, scale bar = 100 µm). Right: Quantification active‐β‐catenin mean fluorescence intensity. H) Lactate concentration in culture medium following 10^6^ CFU *L. amylovorus* treatment of intestinal organoids (n = 4 wells per group). I) Correlation analysis between proliferation levels and Wnt/β‐catenin signaling pathway in organoids with lactate concentration in culture medium supernatant. J) Lactate concentration in culture medium following different doses of *L. amylovorus* treatment of intestinal organoids (n = 4 wells per group). Graphs represent mean ± SEM. The one‐way ANOVA and multiple comparisons in Fisher's LSD test were performed while asterisks mean statistically significant difference: *P ≤ 0.05, **P ≤ 0.01, ***P ≤ 0.001, *P* ＞ 0.05 no significance (ns).

In contrast to live *L. amylovorus* treatment, heat‐killed *L. amylovorus* failed to induce organoid expansion and proliferation, and increase mRNA abundance of aISC marker genes and Wnt/β‐catenin related genes, indicating live bacteria products are needed to drive changes in the intestinal epithelium (**Figure** **3**A–G). Because one of the major metabolites produced by *Lactobacillus* is lactate, we measured its concentration in organoid culture media following treatment. Live *L. amylovorus* (*P* = 0.0001) and *L. salivarius* (*P* < 0.0001) treatment both increased lactate concentration in organoid culture media, which was not observed after treatment with heat‐killed *L. amylovorus* (*P* = 0.583) (Figure 3H; Figure S5H, Supporting Information). Interestingly, the *Lactobacillus* species, *L. mucosae*, only marginally increased organoid growth and Wnt signaling, and only modestly increased lactate concentration in culture media. In addition, lactate concentration in the culture supernatants was positively correlated the jejunal organoids proliferation status and the mRNA expression of Wnt/β‐catenin signaling (Figure 3I). These data strongly suggest a possibility that *Lactobacillus* affects intestinal epithelial homeostasis through lactate production.

### 2.5 Lactate Promotes Porcine Jejunal Organoid Proliferation Through Activating Wnt/β‐catenin Signaling

**Figure 4:**
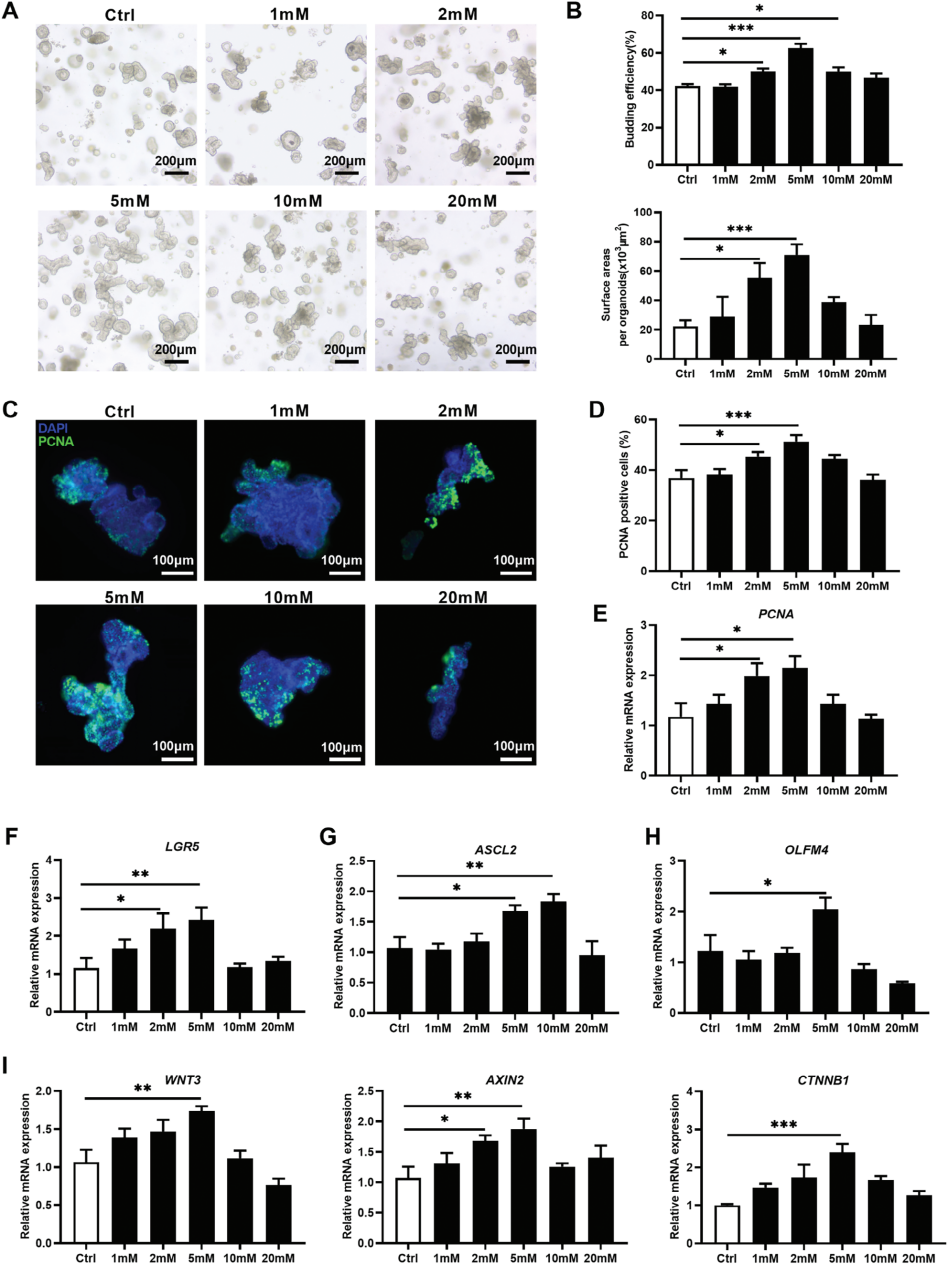
Dose dependent effect of lactate on porcine jejunal organoid proliferation and Wnt/β‐catenin signaling‐related genes. A) 1, 2, 5, 10, and 20 mM of lactate were used to treat organoids for 48 h (n = 5 wells per group). Organoid morphology was assessed by light microscopy (Scale bar = 200 µm). B) Quantification of the budding efficiency and the organoid area (n = 5 wells per group). C) Representative images of immunofluorescent imaging of PCNA stained jejunal organoids (green: PCNA, blue: DNA, scale bar = 100 µm, n = 5 wells per group). D) Quantification of PCNA positive‐cells percentage (n = 5 wells per group). E) Relative mRNA abundance of *PCNA* in organoids. F–H) Relative mRNA expression of aISC markers including *LGR5*, *ASCL2*, and *OLFM4* in jejunal organoids (n = 5 wells per group). I) Relative mRNA expression of Wnt/β‐catenin signaling‐related genes *WNT3*, *AXIN2*, and *CTNNB1* in organoids (n = 5 wells per group). Graphs represent mean ± SEM. The one‐way ANOVA and multiple comparisons in Fisher's LSD test were performed while asterisks mean statistically significant difference: *P ≤ 0.05, **P ≤ 0.01, ***P ≤ 0.001.

We subsequently tested how *Lactobacillus*‐produced lactate may contribute to intestinal homeostasis in a dose‐dependent manner. A total of five doses of lactate, including 1 mM, 2 mM, 5 mM, 10 mM, and 20 mM, were used to treat intestinal organoids. The doses were selected based on the increased levels of lactate observed in the culture supernatant after treating the organoids with different doses of live *L. amylovorus*, compared to the mock‐treated group (Figure 3J). At these doses, treatment of intestinal organoids with 2 mM and 5 mM lactate for 48 h significantly increased the growth and proliferation of intestinal organoids relative to mock‐treated organoids (**Figure** **4**A–D). Subsequent RT‐qPCR analysis showed that 2 mM lactate significantly increased *PCNA* (*P* = 0.045), *LGR5* (*P* = 0.041), and *AXIN2* (*P* = 0.038) (Figure 4E,F,I). Only the treatment with 5 mM lactate, reflecting increased lactate concentration observed following treatment with 10^6^ live *L. amylovorus*, resulted in increased mRNA abundance of proliferation marker *PCNA* (*P* = 0.045), aISC markers including *LGR5* (*P* = 0.01), *ASCL2* (*P* = 0.030) and *OLFM4* (*P* = 0.017), and Wnt/β‐catenin signaling related genes *WNT3* (*P* = 0.0017), *AXIN2* (*P* = 0.0042), and *CTNNB1* (*P* < 0.001) (Figure 4E–I). At dose of 1 mM, 10 mM, and 20 mM, lactate had no significant effects on porcine intestinal organoids proliferation and Wnt/β‐catenin signaling (Figure 4A–E,I). These findings indicate that lactate stimulates porcine jejunal organoids through activating Wnt/β‐catenin signaling in a dose‐dependent manner, similar to *Lactobacillus‐*induced alterations of organoids. These results suggest that *Lactobacillus* alters intestinal epithelial homeostasis through its metabolite lactate.

### 2.6 Lactate Promotes Organoid Growth in a GPR81‐Dependent Manner

**Figure 5:**
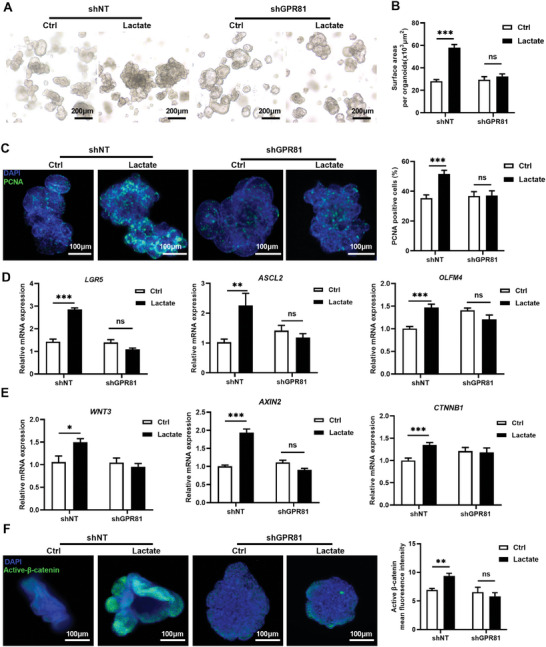
GPR81 mediates lactate‐induced ISC proliferation and Wnt/β‐catenin signaling. A) 5 mM lactate was used to treat intestinal organoids with (shGRP81) or without GPR81 (shNT) knockdown for 48 h. Organoid morphology was assessed by light microscopy (Scale bar = 200 µm). B) Quantification of organoid area (n = 5 wells per group, 15 organoids per well). C) Immunofluorescent imaging of PCNA stained porcine jejunal organoids. Left: representative images (green: PCNA, blue: DNA, scale bar = 100 µm, n = 5 wells per group). Right: Quantification percentage of PCNA‐positive cells (15 organoids per well). D) Relative mRNA expression of *LGR5*, *ASCL2*, *OLFM4* in jejunal organoids. E) Relative mRNA expression of Wnt/β‐catenin signaling related genes *WNT3*, *AXIN2* and *CTNNB1* in organoids (n = 5 wells per group). F) Immunofluorescent imaging of Active‐β‐catenin stained porcine jejunal organoids. Left: representative images (green: Active‐β‐catenin, blue: DNA, scale bar = 100 µm, n = 5 well per group). Right: Quantification active‐β‐catenin mean fluorescence intensity. Graphs represent mean ± SEM. The Student's *t*‐test was performed between two groups while asterisks mean statistically significant difference: *P ≤ 0.05, **P ≤ 0.01, ***P ≤ 0.001, *P* ＞ 0.05 no significance (ns).

Because GPR81 is a major receptor for lactate, it is likely that lactate produced by *Lactobacillus* acts on this receptor to regulate intestinal epithelial growth. We first measured the expression of GPR81 in porcine intestinal epithelium in vivo and in vitro. RT‐qPCR studies showed that *GPR81* mRNA expression was higher in Meishan pigs (*P* = 0.003, Figure S6A, Supporting Information). Immunofluorescence staining illustrated in both breeds, GPR81 was expressed primarily in crypts, and Meishan pigs had higher GPR81 staining intensity (*P* = 0.039, Figure S6B, Supporting Information). Furthermore, studies using porcine jejunal epithelial organoids showed lactate treatment increased *GPR81* mRNA expression (*P* = 0.016, Figure S8A, Supporting Information). Thus, GPR81 may be responsible for lactate‐induced porcine jejunal proliferation. To test this, *GPR81* was effectively knocked down using a lentiviral system in intestinal organoids (Figure S7A and B, Supporting Information). GFP expression confirmed the successful lentiviral transduction in intestinal organoids (Figure S7A, Supporting Information). RT‐PCR further confirmed a significant (≈50%) reduction in *GPR81* mRNA abundance in the intestinal organoids of the shRNA‐GPR81 (shGPR81) group compared to the negative control (shNT) group (*P* < 0.001) (Figure S7B, Supporting Information). 5 mM lactate significantly increased organoid size (*P* < 0.001) and percentage of PCNA‐positive cells (*P* = 0.001) of intestinal porcine organoids in the shNT group, but had no impact on intestinal porcine intestinal organoids in the shGPR81 group (**Figure** **5**A–C). Similar to the growth and proliferation of intestinal organoids presented above, the dose of 5 mM lactate only increased mRNA for the aISC markers including *LGR5* (*P* < 0.0001), *ASCL2* (*P* = 0.0031) and *OLFM4* (*P* = 0.0002) (Figure 5D), and Wnt/β‐catenin signaling related genes *WNT3* (*P* = 0.0143), *AXIN2* (*P* < 0.0001), and *CTNNB1* (*P* = 0.006) (Figure 5E), as well as β‐catenin activity in the shNT group (*P* < 0.0037) (Figure 5F).

GPR81 activity could be modulated by well‐established GPR81 agonist 3,5‐DHBA (3‐chloro‐5‐dihydroxybenzoic acid) and GPR81 antagonist 3‐OBA (3‐hydroxy‐butyrate).^[^ ^26^, ^27^, ^28^ ^]^ Treatment with lactate (*P* = 0.005) or 3,5‐DHBA (*P* = 0.003) increased organoid size compared to mock treatment, while 3‐OBA inhibited lactate‐induced jejunal organoid size change (*P* = 0.031) (Figure S8B and C, Supporting Information). Similar to experiments presented above, treatment with lactate or 3,5‐DHBA increased proliferation marker, aISC marker, and Wnt/β‐catenin signaling‐related gene expression (Figure S8D–H, Supporting Information), and lactate‐induced changes were blocked by 3‐OBA treatment (Figure S8D–H, Supporting Information). These results demonstrate that GPR81 activation alone can promote intestinal epithelial proliferation and β‐catenin activation, and lactate‐induced intestinal epithelial changes are diminished by GPR81 blockade. Together, these data show that lactate regulates ISC function through GPR81.

### 2.7 Knockdown of GPR81 Diminishes *L. amylovorus*‐Induced Organoid Growth

**Figure 6:**
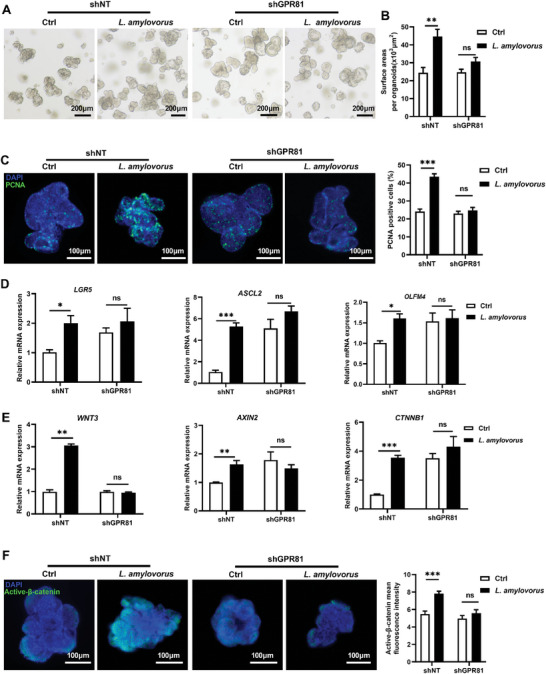
Inhibiting effects of knockdown GPR81 on the proliferation of porcine intestinal organoids and activation of Wnt/β‐catenin signaling promoted by *L. amylovorus*. A) 10^6^ CFU *L. amylovorus* was used to treat intestinal organoids with (shGRP81) or without (shNT) GPR81 knockdown for 48 h. Organoids morphology was assessed by light microscopy (Scale bar = 200 µm). B) Quantification of organoid area (n = 5 wells per group, 15 organoids per well). C) Immunofluorescent imaging of PCNA stained porcine jejunal organoids. Left: representative images (green: PCNA, blue: DNA, scale bar = 100 µm, n = 5 wells per group). Right: Quantification percentage of PCNA‐positive cells (15 organoids per well). D) Relative mRNA expression of *LGR5*, *ASCL2*, *OLFM4* in jejunal organoids. E) Relative mRNA expression of Wnt/β‐catenin signaling related genes *WNT3*, *AXIN2* and *CTNNB1* in organoids (n = 5 wells per group). F) Immunofluorescent imaging of Active‐β‐catenin stained porcine jejunal organoids. Left: representative images (green: Active‐β‐catenin, blue: DNA, scale bar = 100 µm, n = 5 well per group). Right: Quantification active‐β‐catenin mean fluorescence intensity. Graphs represent mean ± SEM. The student's *t*‐test was performed between two groups while asterisks mean statistically significant difference: *P ≤ 0.05, **P ≤ 0.01, ***P ≤ 0.001，*P* ＞ 0.05 no significance (ns).

These results above demonstrated that lactate from *L. amylovorus* regulates jejunal organoid growth, and that GPR81 is responsible for lactate‐induced organoid expansion. This points to the possibility that *L. amylovorus* controls intestinal epithelial cell growth through a GPR81‐dependent mechanism. To test the hypothesis, *L. amylovorus* was further treated with porcine jejunal organoids with or without GPR81 knockdown. *L. amylovorus*‐induced effects, including the growth of intestinal organoid (**Figure** **6**A–C), the elevation of aISC markers (*LGR5* [*P* < 0.043], *ASCL2* [*P* < 0.0001] and *OLFM4* [*P* = 0.0152] (Figure 6D), Wnt/β‐catenin related gene expressions (*WNT3* [*P* < 0.0001], *AXIN2* [*P* = 0.0092], and *CTNNB1* [*P* = 0.0005] (Figure 6E), and β‐catenin activity (*P* = 0.0004) (Figure 6F), were diminished by GPR81 knockdown.

To further validate the essential role of GPR81 in promoting the growth of intestinal organoids by *L. amylovorus, L. amylovorus*‐treated organoids were further treated with the GPR81 antagonist 3‐OBA. Both *L. amylovorus* alone (*P* = 0.0002) and *L. amylovorus* plus 3‐OBA treatment (*P* = 0.0004) increased culture media lactate concentration, and there was no difference in lactate concentration between groups (Figure S9A, Supporting Information), indicating GPR81 inhibition does not prevent *L. amylovorus*‐induced lactate production in culture media. In contrast, 3‐OBA treatment (*P* = 0.002) blocked *L. amylovorus*‐induced enlargement of jejunal organoids (*P* = 0.003) (Figure S9B and C, Supporting Information). 3‐OBA diminished organoid proliferation (Figure S9D and E, Supporting Information), and increased mRNA for aISC markers (Figure S9F, Supporting Information), Wnt/β‐catenin related gene expression, and β‐catenin activity (Figure S9G and H, Supporting Information). These results suggest that the proliferative effect of *L. amylovorus* on porcine jejunal organoids is primarily mediated through a GPR81‐dependent mechanism.

### 2.8 *L. amylovorus*‐Lactate‐GPR81 Axis Regulates Porcine Jejunal Organoid Differentiation

We further explored the effect of the *L. amylovorus*‐lactate‐GPR81 axis on porcine jejunal organoid differentiation (Figure S10, Supporting Information). Treatment with 10^6^ CFU of *L. amylovorus* or 5 mM lactate significantly increased mRNA expression of Paneth cell marker (*LYZ1*) (*P* = 0.0034 and *P* = 0.0198, respectively) and Tuft cell marker (*DCLK*) (*P* = 0.0276 and *P* < 0.001, respectively), decreased mRNA expression of enterocyte marker (*FABP*) (*P* = 0.0013 and *P* = 0.0217, respectively) compared to mock‐treated organoids in negative control (shNT) group, while this treatment had no noticeable effect on intestinal organoids in the shGPR81 group (Figure S10A–D,I,J, Supporting Information). *L. amylovorus* and lactate had no effect on the mRNA expression of goblet cell (*MUC2*) (*P* = 0.977 and *P* = 0.8285, respectively) (Figure S10E and G, Supporting Information). In addition, *L. amylovorus* (*P* = 0.0030), rather than lactate (*P* = 0.6812), increased mRNA expression of enteroendocrine cell marker (*CHGA*) in porcine intestinal organoids of control group, which is abrogated by GRP81 knockdown (Figure S10F and H, Supporting Information). These findings suggest that the *L. amylovorus‐*lactate‐GPR81 axis promotes the differentiation of secretory lineages such as Paneth cell, Tuft cell.

## 3 Discussion

Employing pigs with genetically distinct phenotype, we uncover a breed‐related variation in the small intestinal microbiota, which is characterized by the higher *Lactobacillus* (e.g., *L. amylovorus*) abundance and lactate‐producing gene expression network in an obese breed than lean breed. These differences are associated with increased intestinal aISC function, and Wnt/β‐catenin signaling. By using porcine jejunal organoids, we further prove that live *L. amylovorus* and its metabolic product lactate promote intestinal epithelial proliferation, aISC function, and Wnt/β‐catenin signaling. Our studies also show *L. amylovorus* and lactate exert these effects through the lactate receptor GPR81. These results suggest that a *Lactobacillus‐*lactate‐GPR81‐Wnt/β‐catenin signaling axis exists to drive ISC function to promote intestinal epithelial proliferation. Taken together, this study suggests the relevance of phenotype variations with intestinal microbiome and highlights the importance of *Lactobacillus* species on ISC function in the small intestine.

Multiple studies have shown that host genetic factors affect intestinal microbial composition and function.^[^ ^11^, ^29^, ^30^ ^]^ We first determined if two breeds of pigs with different phenotypes harbor distinct microbiome. In this study, both breeds of pigs were housed in different sections of the same pigsty with the same diet for 28 days, with only metal guardrails separated for these strains, allowing significant sharing of the environmental microbiome. Thus, environmental factors are largely identical in these breeds, allowing proper comparison of microbial composition.

Our results show that obese Meishan pigs have higher abundance of *Lactobacillus* (such as *L. amylovorus*). The relationship between body fat content and *Lactobacillus* composition could be a generalized finding, as a study comparing ileal digesta microbiome between the obese Chinese Shaziling breed and the lean western European Yorkshire breed also identified higher *Lactobacillus* abundance in Shaziling pigs.^[^ ^31^ ^]^ In addition, some human studies also associated obesity with *Lactobacillus* abundance.^[^ ^32^, ^33^ ^]^

Employing metatranscriptomics profiling of small intestinal content, the present study further uncovers *Lactobacillus* as dominant genus producing lactate‐metabolizing enzymes and lactate in pigs. *Lactobacillus* is one of the core genera inhabiting the swine small intestine across the growth stages of pigs.^[^ ^34^ ^]^ Some studies have found that *Enterococcus* is primarily responsible for lactate production in human small intestine.^[^ ^35^ ^]^ These results may represent the uniqueness of microbial ecology in the small intestine of pigs relative to human.

In addition to gut microbiome differences, histological analysis shows the two distinct breeds have different jejunal tissue organization. Meishan pigs have higher crypt depth, and this is associated with increased crypt proliferation, ISC number, and Wnt/β‐catenin signaling activity. These data suggest breed difference in ISC function. Despite these results, it is not clear if these in vivo results are connected. It has been reported in mice that high‐fat diet‐induced obesity leads to increased crypt depth and ISC number.^[^ ^36^, ^37^ ^]^ *Lactobacillus*, including *L. amylovorus*, has been used as probiotics.^[^ ^38^ ^]^ As demonstrated previously, certain *Lactobacillus* species can promote ISC function to promote epithelial damage repair in mouse intestinal epithelial organoids.^[^ ^4^, ^17^, ^27^ ^]^ Furthermore, it is recognized that lactate can benefit small intestinal health, by promoting bacterial colonization resistance and modulation of host intestinal immunity and epithelial development.^[^ ^27^, ^39^ ^]^ Taken together, these findings demonstrate a potential pathway for lactate‐producing bacteria to regulate ISC function.

The inner epithelium lining the small intestine has multiple tasks, including nutrient digestion and absorption, balancing water and ion secretion and absorption, and reliable pathogen resistance.^[^ ^2^ ^]^ These cells face a harsh intestinal luminal environment, which necessitates continuous intestinal self‐renewal, that is driven by ISCs located in the intestinal crypts.^[^ ^40^ ^]^ The gut microbiome in the small intestine has been increasingly recognized as an important regulator in intestinal homeostasis, such as barrier function and nutrient absorption.^[^ ^41^, ^42^ ^]^ Although it has been reported that gut microbiome can regulate ISC function, our understanding of such regulation remains rudimentary. Thus, understanding how gut microbiome regulates ISC function remains a high research priority.

*Lactobacillus* is a dominant genus in the porcine small intestine,^[^ ^43^ ^]^ and we identify that its abundance is significantly higher in Meishan pigs, along with increased ISC function. However, this link is difficult to dissect in vivo due to physiological complexity. Thus, we tested the potential linkage between these events by using intestinal organoid models, which allowed us to perform detailed morphology and molecular analyses.^[^ ^4^, ^44^, ^45^ ^]^ Our study demonstrates that live *L. amylovorus* promotes ISC function via a lactate‐GPR81‐Wnt/β‐catenin signaling. In combination with in vivo findings that Meishan pigs have high *Lactobacillus* abundance and lactate concentration, ISC activity, and Wnt/β‐catenin signaling activity, these results indicate that Meishan pigs exert higher ISC function in the jejunum through *Lactobacillus*.

There are several limitations in this study. Two pig breeds (Meishan and Yorkshire) were employed in the present study. It is not clear whether such results can be generalized in other pig breeds with distinct body fat content, or can be generalized to large omnivorous mammals, including human. This study mainly focused on the effect of the gut microbiome on ISC function; however, whether the intestinal epithelial proliferation and ISC changes affect barrier function, nutrient absorption, and organismal metabolism to drive body fat content remains to be investigated.

## 4 Conclusion

Employing the obese (Meishan) pigs and lean (Yorkshire) pigs, this study reveals that breed‐dependent microbiome heterogeneity, characterized by *Lactobacillus* and particularly *L. amylovorus*, drives the jejunal epithelial proliferation through Wnt/β‐catenin signaling via *Lactobacillus*‐lactate‐GPR81 axis. These results highlight the important role of microbiome‐stem cell interactions in affecting intestinal function due to genetic factors. Identification of such a pathway may provide novel insights into intestinal homeostasis that can be utilized to impact gut health.

## 5 Experimental Methods

### Animals Experiment

The experimental protocol and procedures for the care and treatment of the pigs were approved by the Animal Care and Use Committee of Nanjing Agricultural University (approval number SYXK 2018‐0071).

Six Meishan barrows (≈66 kg) and Six Yorkshire barrows (≈83 kg) in the same physiological stage were raised in the same commercial farm (Jiangsu Province, China) in this experiment. The phrase “same physiological stage” in this study refers to the period when the body weight is 35% of the adult body weight.^[^ ^46^ ^]^ To investigate the effect of breed on the intestinal microbiota, the growth and feeding conditions for both Meishan and Yorkshire pigs were strictly controlled. All Meishan and Yorkshire pigs were raised together in the same facility and fed the same feed for 28 days. All the pigs had ad libitum access to diet and water throughout the experiment, with the diet details listed in Table S3 (Supporting Information). After the 28‐days trial, the middle sections of the jejunum, and the digesta from the jejunum were collected for further experiment.

### Bacterial Stains

The 16S rRNA gene sequence of *L. salivarius* S1, *L. amylovorus* S1, and *L. mucosae* M1 have been submitted to NCBI under the accession number OR054073, MT525371, and OR054072. *L. salivarius* S1, *L. amylovorus* S1, and *L. mucosae* M1 were isolated from jejunal digesta in pigs, purified and identified as previously described.^[^ ^7^ ^]^ They were grown in MRS medium at 37 °C for 12 h. Subsequently, 100 µL of the bacterial suspension was serially diluted and plated on solid MRS medium for growth for 24 h at 37 °C for colony counting. The bacterial suspension was then centrifuged at 3000 rpm for 10 min to remove the MRS medium, washed, and diluted in advanced DMEM/F12 based on the plate counting results for subsequent treatment of organoids.

### Porcine Jejunal Crypt Isolation and Intestinal Organoid Culture

Porcine jejunal crypts were isolated and cultured according to the described previously protocols, with minor modifications.^[^ ^4^, ^25^, ^47^ ^]^ Briefly, the jejunum was isolated from 3‐mouths‐old Yorkshire pigs, and the jejunum was dissected longitudinally and removed the intestinal contents and mucus with cover slips, and then the jejunum was cut into 1 × 1 cm pieces. Next, the jejunal pieces were washed several times with ice‐cold PBS until the supernatant was clear, and these pieces were incubated and rocked in 10 mM EDTA for 30 min at 4 °C to disassociate the crypts. Crypts enriched in the supernatant were passed through a 70 µm filter and centrifuged at 400 *g* at 4 °C for 5 min. Then intestinal crypts were resuspended and washed with PBS contained with 1% FBS to remove EDTA. After counting, the IntestiCult organoid growth medium (Stemcell) and Matrigel (BD Biosciences) were mixed to resuspend the intestinal crypts, and 50 µL mixture was added into each well in a 24‐well plate. The plate contained with crypts was incubated at 37 °C for 30 min until the Matrigel solidified, and then 500 µL of IntestiCult organoid growth medium was added to each well. Organoids were subsequently cultured in incubators at 37 °C with 5% CO_2_, and the culture medium was completely replenished every 3 to 4 days.

### Treatments, Observations, and Measurements of Intestinal Organoids

Following intestinal organoids passage, *Lactobacillus. spp* was added into the culture medium of intestinal organoids to establish the *Lactobacillus*‐organoids co‐culture model. Different doses of *L. amylovorus* S1 (10^3^, 10^4^, 10^5^, 10^6^, 10^7^ CFU per well) were used to treat intestinal organoids to determine dose dependency treatment. Different doses of lactate were mixed with the IntestiCult organoid growth medium (Stemcell) to prepare organoids culture medium containing 1, 2, 5, 10, and 20 mM lactate. After organoid passage and Matrigel solidification, 500 µL of IntestiCult organoid growth medium containing lactate was added to each well.

The surface area of intestinal organoids was measured according to a previous study.^[^ ^4^ ^]^ Briefly, several random nonoverlapping pictures were acquired from each well using a Zeiss 710 laser scanning confocal microscope. Organoids that were at the edge of the picture and not photographed completely were excluded from the counting. Organoid perimeters for area measurements were defined manually using the Analyze Particle function of ImageJ software (National Institutes of Health).

### Lentiviral Transduction‐Based GPR81 Knockdown

Porcine jejunal organoids were cultured in organoid growth medium to maintain stemness. These organoids were then digested into single cells using TrypLe Express (Gibco), and infected with lentiviral particles (MOI = 10) carrying either a control shRNA (shNT) or GPR81‐specific shRNA (shGPR81) (Shanghai Geneche Co., Ltd.) for a duration of 6 h using HitransGA (Shanghai Geneche Co., Ltd.). Following infection, virus‐containing media was removed via washing, and 5000 cells were plated in Matrigel per well. After 72 h of lentiviral infection of the intestinal organoids, infection efficiency was observed using fluorescence microscopy. The infected porcine jejunal organoids were subsequently passaged using TrypLE Express (Gibco) and plated in Matrigel for subsequent experiments. After 24 h, the dose of 10^6^ CFU *L. amylovorus* or 5 mM lactate was used to treat with intestinal organoids. GPR81 knockdown efficiency was determined by RT‐PCR.

### Western Blot Analysis

Jejunum tissues isolated from the Meishan and Yorkshire pigs and porcine intestinal organoids were lysed in radioimmunoprecipitation assay (RIPA) buffer (Beyotine) containing a protease inhibitor cocktail (Beyotine). BCA protein quantification kit (Beyotine) was used to quantify protein concentrations. 5X SDS‐PAGE loading buffer was added to protein samples to denature the protein at 100 °C for 5 min. 20 µg protein from each sample was separated by SDS‐PAGE with electrophoresis and then the proteins on gels were electrotransferred onto polyvinylidene fluoride (PVDF) membranes (Millipore) for immunoblotting. The following antibodies were used: rabbit anti‐LGR5 (Abclonal, 1:1000), rabbit anti‐Lysozyme (Bioss, 1:1000), mouse anti‐PCNA (Abcam, 1:1000), rabbit anti‐β‐actin (Bioss, 1:1000), goat anti‐rabbit secondary antibodies (Bioss, 1:5000), goat anti‐mouse secondary antibodies (Bioss, 1:5000). Signals were detected using enhanced chemiluminescence (ECL) kits (Beyotine) and a chemiluminescence/fluorescence image analysis system (Tanon). The grayscale of the strip was further analyzed with Image J software.

### Histological Analysis, Immunohistochemistry (IHC), Immunofluorescence (IF), and UEA‐1 Lectin Staining

Two‐centimeter sections of jejunum were collected from each pig in the Yorkshire group and the Meishan group, and then fixed with 4% paraformaldehyde overnight. After embedding in paraffin, samples were serially sectioned at 5 µm thickness and stained with hematoxylin andeosin (HE, Beyotime). Finally, all sections were observed and photographed under an optical microscope (Olympus) and villus height and crypt depth of the jejunum were measured by Image J software.

For immunofluorescence staining and UEA‐1 lectin staining, jejunal tissue sections were deparaffinized in xylene and rehydrated in decreasing concentrations of ethanol. Antigen retrieval was performed by using sodium citrate antigen retrieval Solution (Solarbio, 1:50) for 10 min at 100 °C. Tissue sections were permeabilized with 0.4% Triton X‐100 for 30 min, washed with PBS five times for 5 min each time, and then blocked with 5% bovine serum albumin (BSA) for 2 h. Tissue sections and porcine intestinal organoids were incubated with mouse anti‐PCNA (Abcam, 1:200), rabbit anti‐active β‐catenin (Cell Signaling Technology, 1:200), rabbit anti‐GPR81(Proteintech,1:200), UEA‐1(Sigma, 1:200) overnight at 4 °C, samples were washed with PBS three times and then incubated with an Alexa Fluor 594 or Alexa Fluor 488‐conjugated goat anti‐rabbit antibody(1:250, Bioss) for 60 min at room temperature. 4′,6‐diamidino‐2‐phenylindole (DAPI) was applied for fluorescence staining of DNA in nuclei. All slices were observed and collected under a Zeiss LSM 710 laser scanning confocal microscope for further qualjiushiitative and quantitative analysis. The mean fluorescence intensity of active‐β‐catenin, percentage of PCNA positive cells, and number of UEA‐1 positive cells were analyzed by Image‐Pro Plus software.

For immunohistochemistry, tissue sections were soaked in 3% H_2_O_2_ solution for 10 min after 0.4% Triton X‐100 permeabilization to block endogenous peroxidase activity. Subsequently, the sections were washed with PBS five times for 5 min each time and then blocked with 5% bovine serum albumin (BSA) for 2 h. For Lysozyme staining, sections were incubated with rabbit anti‐lysozyme (Thermo, 1:200) overnight at 4 °C in a humidified box. For the negative control, PBS was used instead of the primary antibody. Following this, the sections were sequentially incubated with biotin‐labeled anti‐rabbit IgG (Boster) and streptavidin‐avidin‐biotin complex (SABC) for 30 min at 37 °C. Finally, the sections were stained using 3, 3′‐diaminobenzidine (DAB) to visualize the positive staining. Lysozyme‐positive cells per crypt in jejunum were measured quantified and analyzed by Image‐Pro Plus software.

### Wnt3a Detection

Jejunum was collected from euthanized pigs and homogenized in ice‐cold PBS containing a protease inhibitor cocktail (Beyotine). Samples were centrifuged at 1200 *g* at 4 °C for 20 min. Supernatants were stored at −20 °C until use for Wnt3a detection. Protein concentrations of the supernatant were determined by using a BCA protein quantification kit (Beyotine). Equal amounts of proteins were used for Wnt3a concentration determination by using a Wnt3a ELISA kit (JiangLai lab) according to the manufacturer's protocol. Absorbance was measured at 450 nm using a spectrophotometer microplate reader (TECAN).

### RNA Extraction and Quantitative Real‐Time PCR

According to the manufacturer's instructions, total RNA was extracted from porcine intestinal organoids or jejunum by RNA iso Plus (Accurate Biotechnology CO., Ltd). RNA was reverse transcribed to synthesize cDNA with a PrimeScript RT Reagent Kit (Accurate Biotechnology CO., Ltd). Realtime PCR was performed by using cDNA as templates and gene‐specific primers. A SYBR green master mix was used for amplification (Accurate Biotechnology CO., Ltd), which was performed by using the QuantStudio 6 Flex System (Applied Biosystems). GAPDH served as the housekeeping gene, and the relative mRNA expression of the target gene was calculated using the 2^−ΔΔ CT^ method. Primers for real‐time RT‐PCR used in this study were listed in Table S4 (Supporting Information).

### Jejunum Digesta Bacterial Composition Analysis

Total genomic DNA was extracted from 0.3 g jejunal digesta using the MagPure Stool DNA kit B (MAGEN) according to manufacturer's instructions. Qubit dsDNA BR Assay kit and 1% agarose gel were used to ensure the concentration and quality of total DNA. The V3‐V4 regions of the bacterial 16S rRNA gene were amplified with universal forward primer (5′‐ACTCCTRCGGGAGGCAGCAG‐3′) and a reverse primer (5′‐GGACTACCVGGGTATCTAAT‐3′).^[^ ^48^ ^]^ Amplicons were purified using an AxyPrep DNA gel extraction kit according to the manufacturer's instructions (Axygen Biosciences). Purified amplicons were used for sequencing on the Illumina MiSeq high‐throughput sequencing platform (BGI), and generating 2 × 300 bp paired‐end reads.

QIIME (version 1.9.1) was used to demultiplex and filter the raw sequencing data. Sequences with an average quality score of more than 20 (accuracy>99%) were retained for downstream analysis. A total of 731,089 high‐quality sequences were acquired, with an average of 60,924 reads per sample. Operational taxonomic units (OTUs) were clustered with a 97% similarity cutoff by using UPARSE (version 7.1) and chimeric sequences were identified and removed using by UCHIME version 4.1.

Taxonomy assignment of OTUs was performed by matching the representative sequences to the 16S rRNA gene database (SILVA version 138) using the Ribosomal Database Project Classifier (version 2.2). Shannon and inverse Simpson diversity indices were calculated with QIIME (http://qiime.org/scripts/split_libraries_fastq.html), along with PCoA based on weighted UniFrac distance. The relative abundance of different bacterial taxa was expressed as percentages.

### Metatranscriptomic Sequencing and Data Analysis

The microbial RNA was extracted from the jejunal content samples using the HiPure stool RNA Kit (MAGEN) according to the manufacturer's instructions. Fragment Analyzer standard sensitivity RNA kits (AATL, DNF‐471) were used to evaluate RNA concentration and quality. Double‐strand cDNA was synthesized after removing genomic DNA and rRNA from fragmented RNA. DNB‐based libraries were constructed with the MGIEASY Small RNA Library Prep Kit V2.0 (MGL) following the manufacturer's instructions. The libraries were sequenced using a MGISEQ‐2000 platform (BGI) and generating paired‐end 100‐bp sequences.

SOAPnuke software was used to trim adapters and remove low‐quality sequences from the raw data. SOAP2 was used to identify and remove host sequences. After removing the reads mapped to the host, we obtained 7748 Mbp of paired‐end sequencing data, with an average of 969 Mbp per sample. High‐quality clean reads from each sample were mapped using bowtie2 to a reference pig gut microbiome gene catalog (PIGC90).^[^ ^49^ ^]^ Only genes that contained at least one mapped read from any of the 8 samples were retained for subsequent analyses.

To align the amino acid sequences of proteins in the mapped gene catalog with Uniprot TrEMBL, DIAMOND (v0.9.21.122) was used, with a threshold of e‐values ≤1e−5. For those genes that were matched to distinguishable taxonomic groups (with multiple records of e‐value ≤1e−5), the taxonomic classification was determined based on the lowest common ancestor algorithms by BASTA (v1.3) at the thresholds of an alignment length >25, identity >80%, and shared by at least 60% of hits. The KEGG (Kyoto Encyclopedia of Genes and Genomes) annotation results were performed with KOBAS (v3.0.3) software (‐t blastout: tab, ‐s ko). The carbohydrate‐active enzymes (CAZymes) were annotated by aligning genes to the dbCAN database (HMMdb V8) with the hmmscan program in HMMER (v3.1b2).

### Measurement of Lactobacillus Population by PCR Assay

Quantities of total bacteria, *Lactobacillus*, *L. amylovorus*, *L. mucosae, L. salivarius*, *L. reuteri*, and *L. delbrueckii* in jejunal digesta were determined by qPCR. The plasmids containing the insert of 16S rRNA genes were constructed using pMD18‐T according to the kit instructions (Takara). The target plasmids were diluted to a series of 10‐fold to generate the standard curves. Realtime PCR was performed according to the previously described method. The copy number of target bacteria in digesta (copy/g) was quantified according to standard curves. The primer sequences of specific 16S rRNA genes were listed in Table S5 (Supporting Information).

### Measurements of Microbial Metabolites

SCFAs in jejunal digesta were measured by liquid chromatography and lactate was measured by gas chromatography according to our previous study.^[^ ^50^, ^51^ ^]^ Lactate concentration in culture medium supermenant of organoids was analyzed using a commercial kit according to the manufacturer's instructions (Nanjing Jiancheng Biological Engineering Institute).^[^ ^52^ ^]^

### Statistical Analysis

Quantitative data were expressed as mean ± SEM, whereas categorical data are expressed as percentages. For normally distributed data sets with equal variances, one‐way ANOVA and multiple comparisons in Fisher's LSD test was employed to determine significant differences among multiple groups, and the student's *t* test was employed to assess differences between the two groups. Differences in bacterial abundance and enzyme abundance for lactate production were analyzed by using the Mann‐Whitney *U* test. *P* < 0.05 was considered statistically significant. All data were visualized by using GraphPad Prism version 9.0.

## Conflict of Interest

The authors declare no conflict of interests.

## Author Contributions

W.Z. conceived, designed, and supervised the study, reviewed and edited the manuscript, and secured funding for the study. H.W. conducted the experimental work, performed statistical tests, and wrote the draft manuscript. C.M. provided advice on the methodology, wrote the draft manuscript, reviewed and edited the manuscript. X.L. analyzed the data related to intestinal microbiota and helped with the manuscript writing. W.F. provided the plasmid of bacteria and *Lactobacillus* train. L.S. supervised the organoid experiments and reviewed and edited the manuscript.

## Supporting information

Supporting Information

## Data Availability

The data that support the findings of this study are available from the corresponding author upon reasonable request.
