# Supplementary material for: Breed‐Driven Microbiome Heterogeneity Regulates Intestinal Stem Cell Proliferation via Lactobacillus‐Lactate‐GPR81 Signaling
Source: Adv Sci (Weinh). 2024 Jun 27;11(33):2400058. doi: 10.1002/advs.202400058 (PMC11434115; doi:10.1002/advs.202400058)
Supplement: Supplementary file 1 — Supporting Information [file ADVS-11-2400058-s001.docx]

**Breed-Driven Microbiome Heterogeneity Regulates Intestinal Stem Cell Proliferation via *Lactobacillus*-Lactate-GPR81 Signaling**

Haiqin Wu^1,2^, Chunlong Mu^3^, Xuan Li^1,2^, Wenlu Fan ^1,2^, Le Shen^4^, Weiyun Zhu* ^1,2^

^1^Laboratory of Gastrointestinal Microbiology, Jiangsu Key Laboratory of Gastrointestinal Nutrition and Animal Health, College of Animal Science and Technology, Nanjing Agricultural University, Nanjing 210095, China

^2^National Center for International Research on Animal Gut Nutrition, Nanjing Agricultural University, Nanjing 210095, China

^3^ Food Informatics, AgResearch, Te Ohu Rangahau Kai, Palmerston North 4474, New Zealand

^4^Department of Surgery, The University of Chicago, Maryland Ave 60637, USA.

*Corresponding author: zhuweiyun@njau.edu.cn, Telephone: +86-25-8439-5523, Fax: +86-25-8439-5314

**Supporting Information**


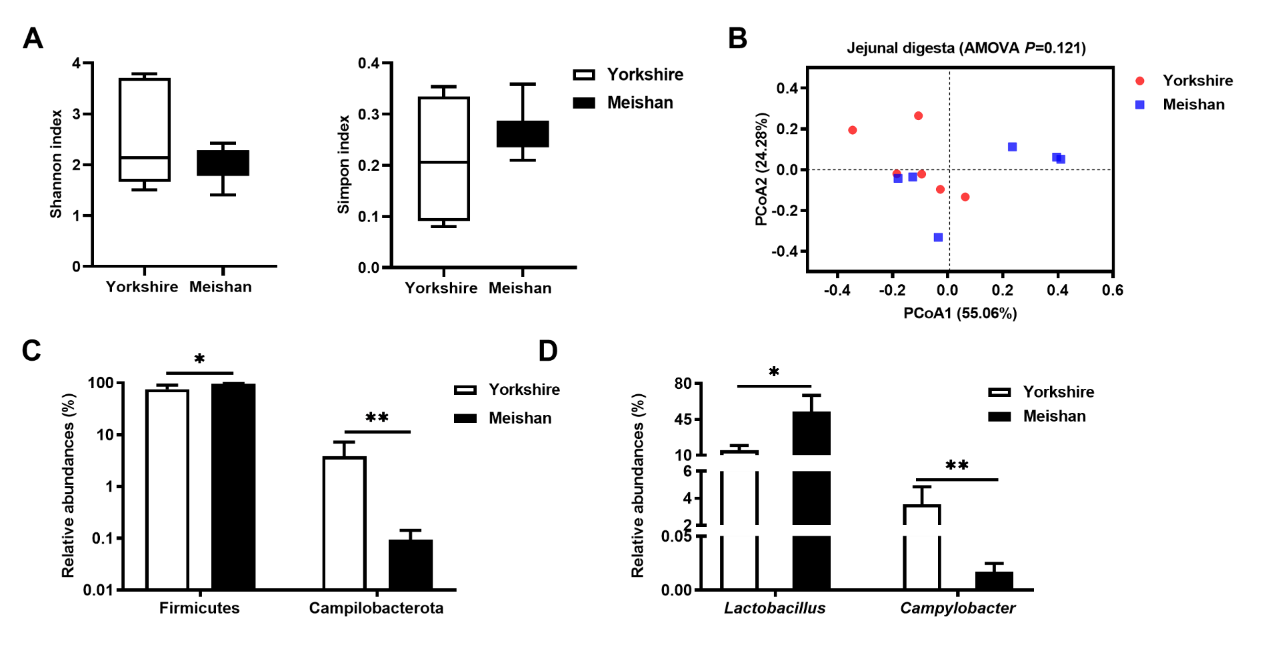


**Figure S1. Microbial diversity, significantly different phyla, genera in the jejunal digesta in Meishan and Yorkshire pigs.**

(A) α-diversity of the microbial community, as determined by Shannon and Simpson indexes (n = 6). The data are expressed as a box-whisker plot. (B) Principal coordinate analysis (PCoA) of jejunal digesta microbiota (n = 6). (C) Significantly different phyla in the jejunal digesta (mean ± SEM, n = 6). (D) Significantly changed genera in the jejunal digesta. Only dominant genera with a mean relative abundance of more than 0.5% in at least one group were enumerated (mean ± SEM, n = 6). Mann-Whitney *U* test was performed between two groups while asterisks mean statistically significant difference: * *P* ≤ 0.05, ** *P* ≤ 0.01.
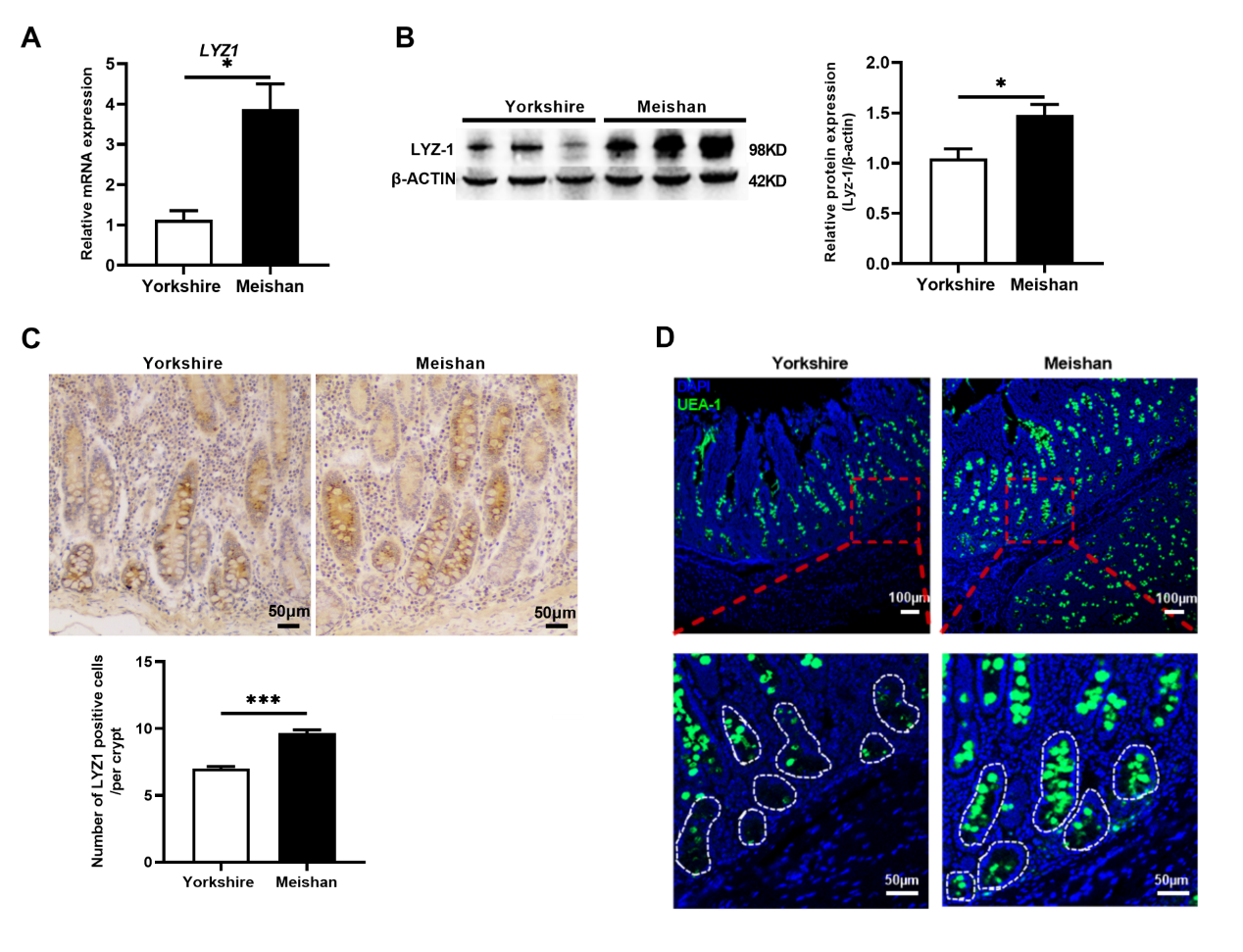


**Figure S2. *LYZ1* mRNA and protein expression, Lysozyme positive cells and UEA-1 positive cells in the jejunum of Meishan and Yorkshire pigs.**

(A) *LYZ1* mRNA expression in the jejunum (n = 6). (B) Western blot analysis of LYZ1 protein expression in jejunal samples. Left panel: representative blot. Right panel: data quantification (n = 3). (C) Immunohistochemical staining of Lysozyme stained jejunal sections (n = 6). Up panel: representative images, down panel: Quantification of Paneth cells number in jejunum crypt. (D) UEA-1 stained jejunal sections (n = 6). Left: representative images (green: UEA-1, blue: DNA). Graphs represent mean ± SEM. The Student's *t*-test was performed between two groups while asterisks mean statistically significant difference: **P* ≤ 0.05, ***P* ≤ 0.01, ****P* ≤ 0.001.


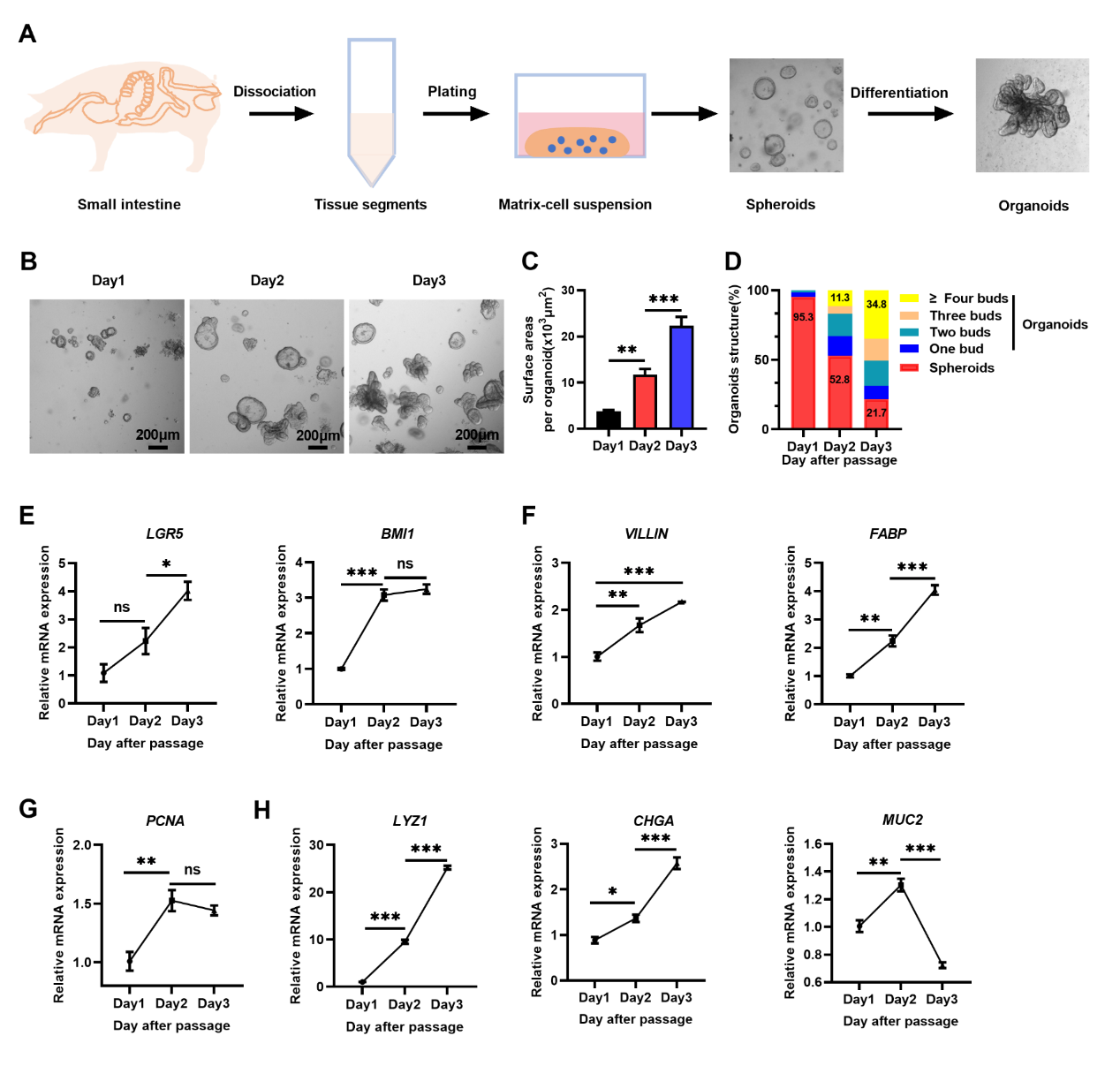


**Figure S3.** **Establishment of porcine intestinal organoids model and analysis of the mRNA expressions of various types of intestinal epithelial marker genes.**

1. Schematic diagram of isolated, culture, and passaging of organoids. (B) Organoid morphology was assessed by light microscopy (Scale bar = 200 μm). (C) The surface areas per organoid were calculated (n = 3 wells per group, 15 organoids per well). (D) The percentage of organoid structure was calculated. (E-H) *LGR5*, *BMI1*, *VILLIN*, *FABP*, *PCNA*, *LYZ1*, *CHGA* and *MUC2* relative mRNA abundance in jejunal porcine organoids (n = 3 wells per group). Error bar represents mean ± SEM. The one-way ANOVA and multiple comparisons in Fisher’s LSD test were performed while asterisks mean statistically significant difference: **P* ≤ 0.05, ***P* ≤ 0.01, ****P* ≤ 0.001, *P* > 0.05 no significance (ns).


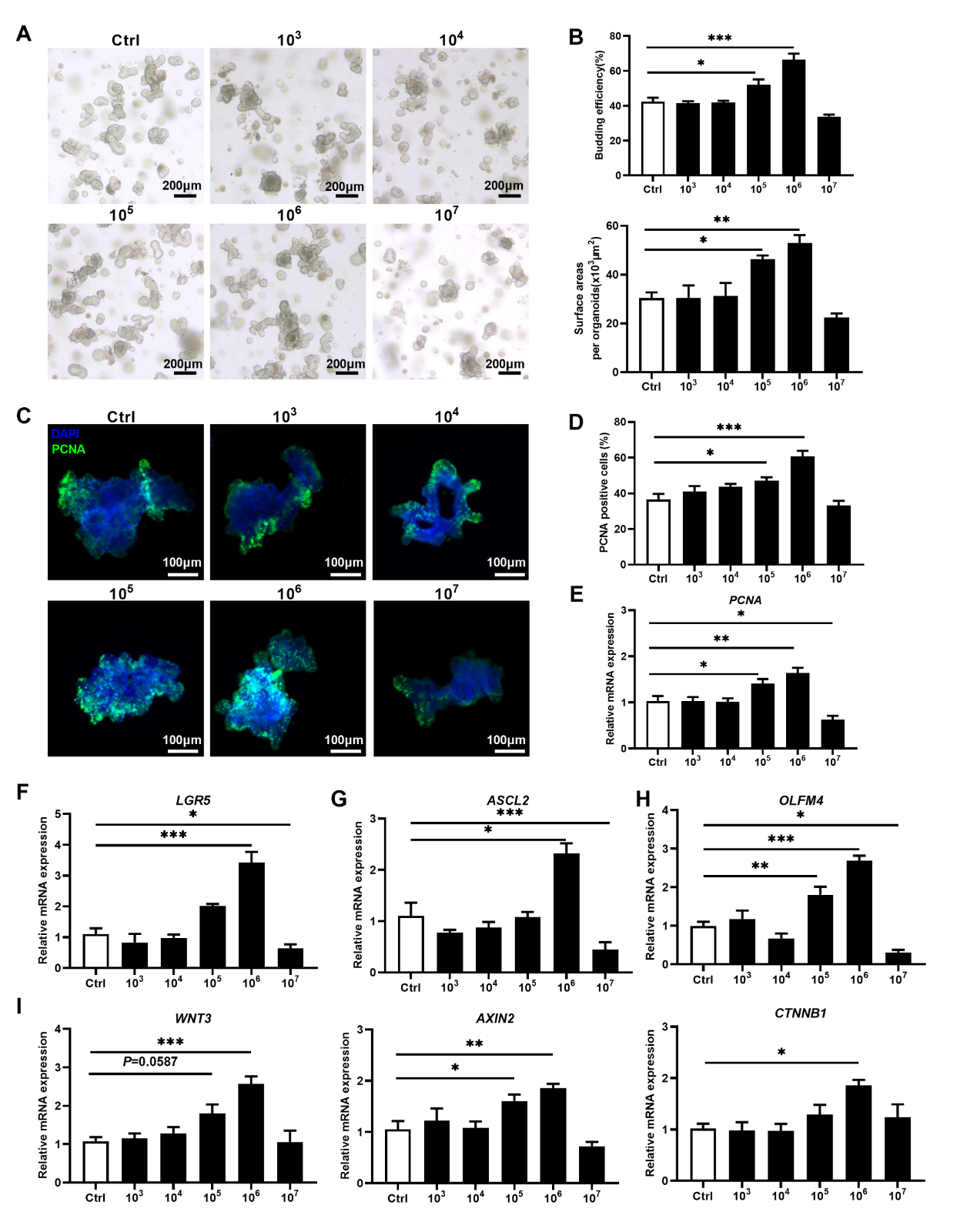


**Figure S4. Dose dependent effects of *L. amylovorus* on porcine jejunal organoid proliferation and Wnt/β-catenin signaling.**

(A) Five doses of *L. amylovorus* including 10^3^, 10^4^, 10^5^, 10^6^, and 10^7^ CFU per well were used to treat organoids for 48 h (n = 5 wells per group). Organoids morphology was assessed by light microscopy (Scale bar = 200 μm). (B) Quantification of the budding efficiency and the organoid area (n = 5 wells per group). (C) Representative images of immunofluorescent imaging of PCNA stained jejunal organoids. (green: PCNA, blue: DNA, scale bar = 100 μm, n = 5 wells per group). (D) Quantification of PCNA positive-cells percentage (n = 5 wells per group). (E) Relative mRNA abundance of *PCNA* in organoids. (F-H) Relative mRNA expression of aISC markers including *LGR5*, *ASCL2,* and *OLFM4* in jejunal organoids (n = 5 wells per group). (I) Relative mRNA expression of Wnt/β-catenin signaling-related genes *WNT3*, *AXIN2,* and *CTNNB1* in organoids (n = 5 wells per group). Graphs represent mean ± SEM. The one-way ANOVA and multiple comparisons in Fisher’s LSD test were performed while asterisks mean statistically significant difference: **P* ≤ 0.05, ***P* ≤ 0.01, ****P* ≤ 0.001.


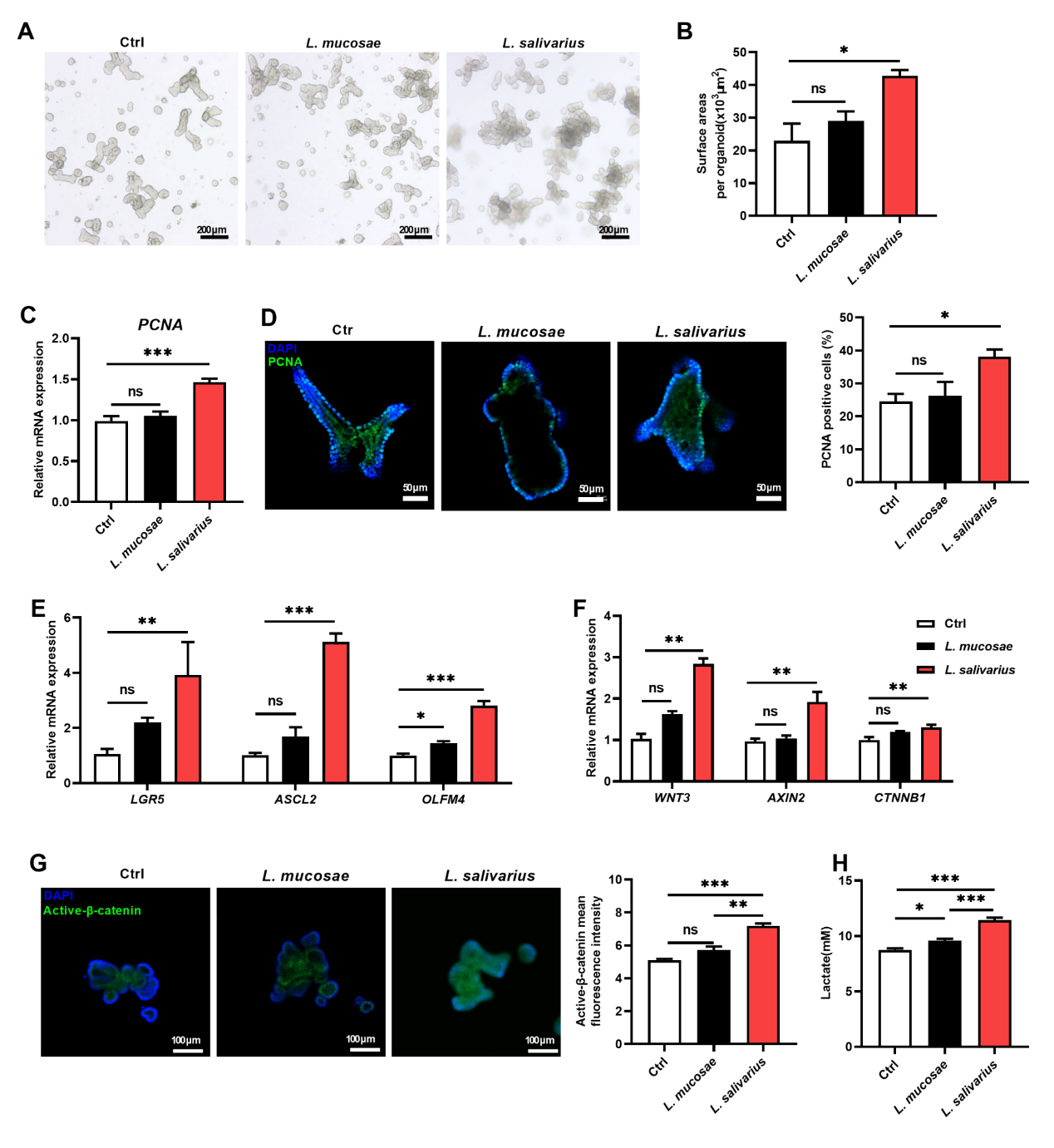


**Figure S5. Effect of *L. mucosae* and *L. salivarius* on porcine jejunal organoid proliferation and marker gene expression.**

(A) 10^6^ CFU *L. mucosae* and *L. salivarius* was used to treat organoids for 48 h respectively (n = 4 wells per group). Organoids morphology was assessed by light microscopy (Scale bar = 200 μm). (B) Quantification of organoid area (n = 4 wells per group, 15 organoids per well). (C) Relative mRNA expression of *PCNA* (n = 4 wells per group). (D) Immunofluorescent imaging of PCNA stained porcine jejunal organoids. Left: representative images (green: PCNA, blue: DNA, scale bar = 50 μm). Right: Quantification percentage of PCNA-positive cells (15 organoids per well). (E) Relative mRNA expression of *LGR5*, *ASCL2* and *OLFM4* in jejunal organoids (n = 4 well per group). (F) Relative mRNA expression of Wnt/β-catenin signaling related genes *WNT3*, *AXIN2* and *CTNNB1* in organoids (n = 4 well per group). (G) Immunofluorescent imaging of Active-β-catenin stained porcine jejunal organoids (n = 4 well per group). Left: representative images (green: Active-β-catenin, blue: DNA, scale bar = 100 μm). Right: Quantification active-β-catenin mean fluorescence intensity. (H) Lactate concentration in the supernatant of porcine jejunal organoids. (n = 4 well per group). Graphs represent mean ± SEM. The one-way ANOVA and multiple comparisons in Fisher’s LSD test were performed while asterisks mean statistically significant difference: **P* ≤ 0.05, ***P* ≤ 0.01, ****P* ≤ 0.001, *P* > 0.05 no significance (ns).


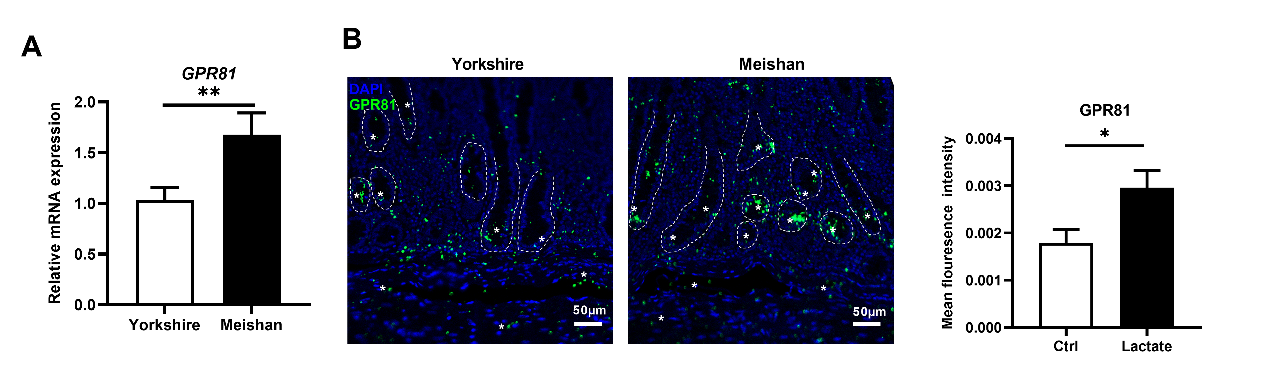


**Figure S6. Localization and expression of GPR81 in the jejunum of Meishan and Yorkshire pigs.**

(A) Relative mRNA expression of *GPR81* in the jejunum (n = 6). (B) Immunofluorescent imaging of GPR81 stained jejunal sections. Left: representative images (green: GPR81, blue: DNA, scale bar = 50 μm). Right: Quantification of mean fluorescence intensity of GPR81 (n = 4). Graphs represent mean ± SEM. The Student's *t*-test was performed between two groups while asterisks mean statistically significant difference: **P* ≤ 0.05, ***P* ≤ 0.01.

**
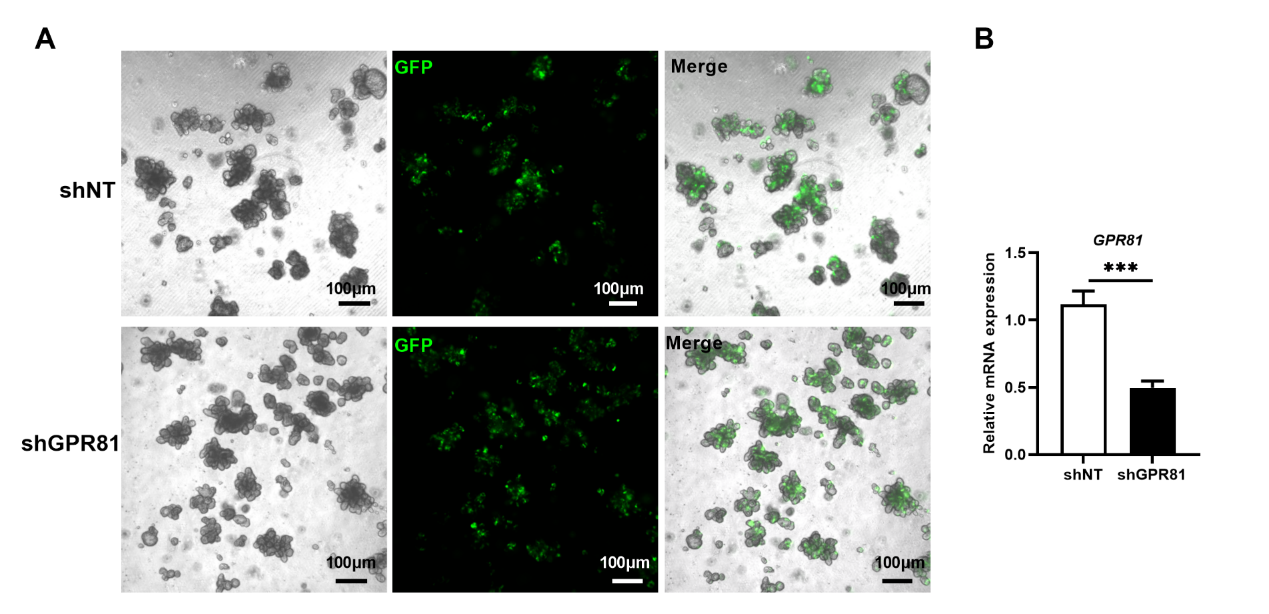
**

**Figure S7. Efficient knockdown of *GPR81* in porcine jejunal organoids.**

(A) The expression of GFP fluorescence in infected intestinal organoids. Representative immunofluorescent images of GPR81-specific shRNA (shGPR81) and control shRNA (shNT)-infected intestinal organoids (green: GFP, scale bar = 100 μm). (B) RT-qPCR verified the knockdown efficiency of *GPR81* (n = 5 per group). Graphs represent mean ± SEM. The Student's *t*-test was performed between two groups while asterisks mean statistically significant difference: **P* ≤ 0.05, ***P* ≤ 0.01, ****P* ≤ 0.001.


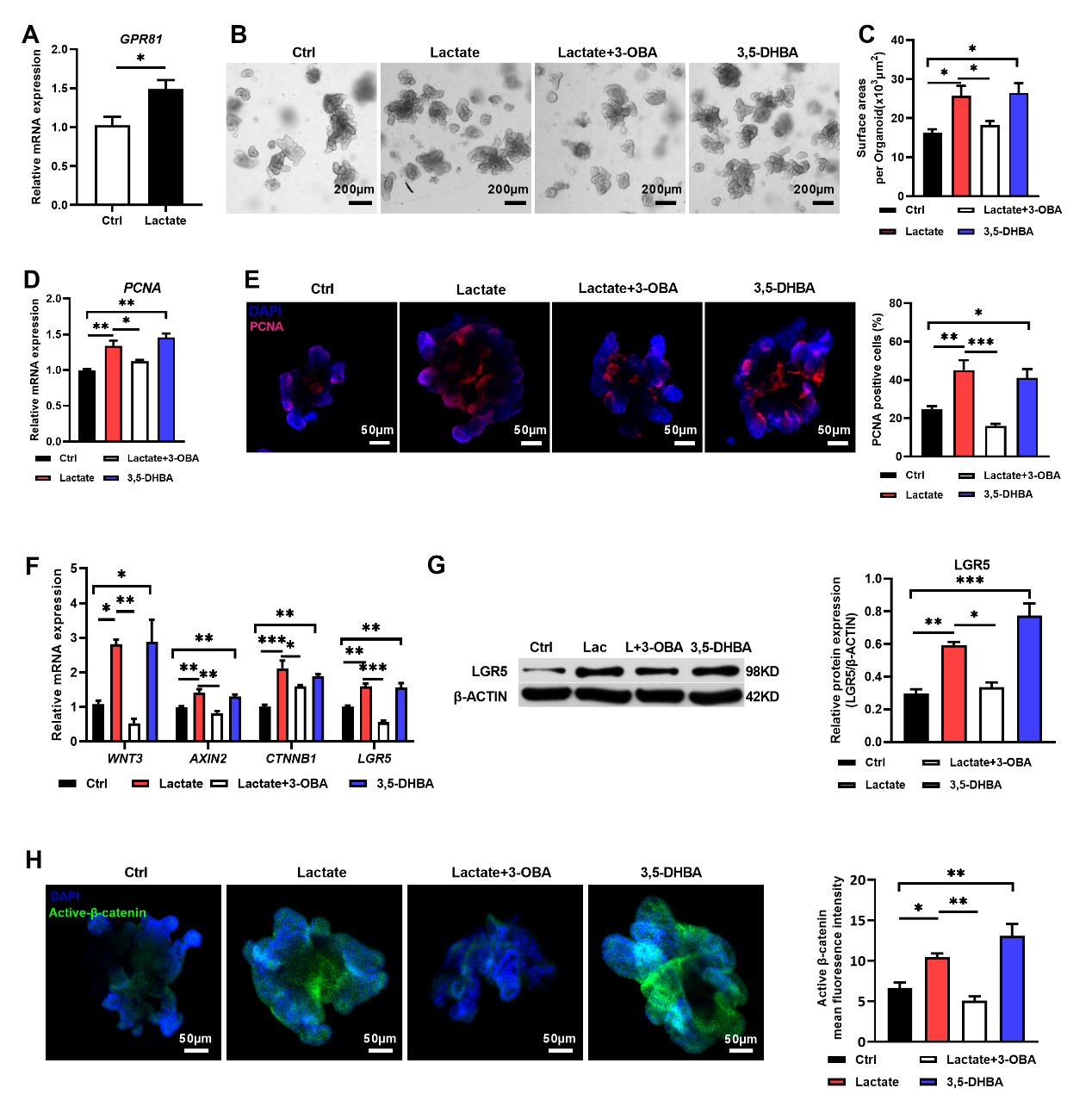


**Figure S8. Effects of GPR81 agonist and antagonist on lactate-induced porcine intestinal organoid proliferation.**

1. Relative mRNA expression of GPR81 in jejunal organoids (n = 6). (B) 5 mM lactate, lactate+3-OBA (GPR81 antagonist) and 3, 5-DHBA (GPR81 agonist) were used to treat organoids (n = 4 wells per group). Organoid morphology was assessed by light microscopy (Scale bar = 200 μm). (C) Surface areas of organoids (n = 4 wells per group, 15 organoids per well). (D) Relative mRNA abundance of *PCNA* in organoids. (E) Immunofluorescent imaging of PCNA stained jejunal organoids. Left: representative images (red: PCNA, blue: DNA, scale bar = 50 μm). Right: Quantification of PCNA positive-cells percentage (n = 4 wells per group). (F) Relative mRNA expression of Wnt/β-catenin signaling related genes *WNT3*, *AXIN2*, *CTNNB1* and *LGR5* in organoids (n = 4 wells per group). (G) Western blot analysis of LGR5 protein expression in organoids (n = 3 wells per group). Left: representative blot. Right: Quantification of LGR5 expression. (H) Immunofluorescent imaging of active-β-catenin stained porcine jejunal organoids. Left: representative images (green: active-β-catenin, blue: DNA, scale bar = 50 μm) (n = 4 wells per group). Graphs represent mean ± SEM. The one-way ANOVA and multiple comparisons in Fisher’s LSD test were performed while asterisks mean statistically significant difference: ** P* ≤ 0.05, ** *P* ≤ 0.01, *** *P* ≤ 0.001.


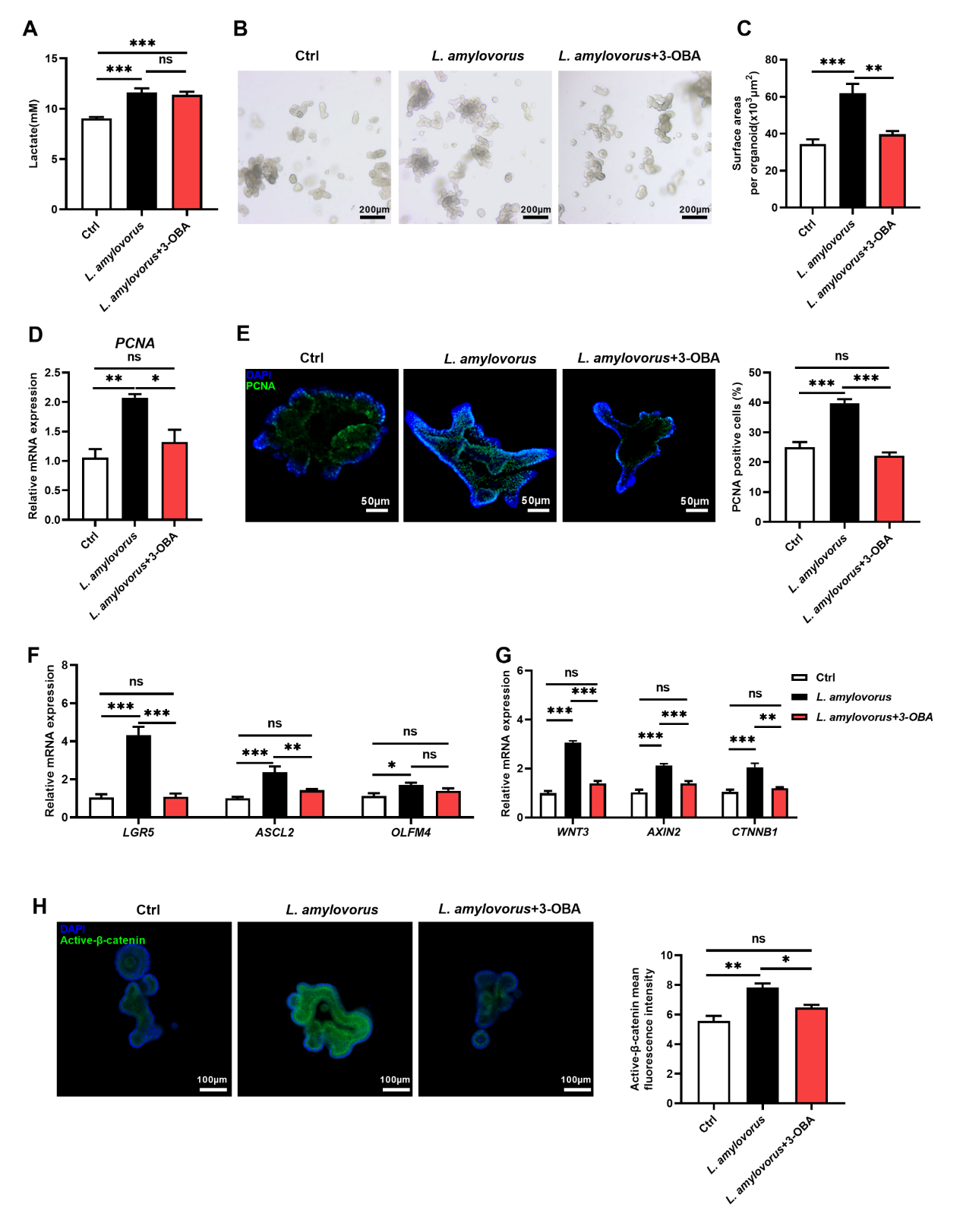


**Figure S9. Blocking effect of 3-OBA on the proliferation of porcine intestinal organoids and activation of Wnt/β-catenin siganling promoted by *L. amylovorus.***

(A) 10^6^ CFU *L. amylovorus*, *and L. amylovorus,* and 3-OBA (GPR81 antagonist) were used to treat organoids respectively for 48 h. Lactate concentration in the supernatant of porcine jejunal organoids (n = 4 wells per group). (B) Organoids morphology was assessed by light microscopy (Scale bar = 200 μm). (C) Quantification of organoid area (n = 4 wells per group, 15 organoids per well). (D) Relative mRNA expression of *PCNA* (n = 4 wells per group). (E) Immunofluorescent imaging of PCNA stained porcine jejunal organoids. Left: representative images (green: PCNA, blue: DNA, scale bar = 50 μm). Right: Quantification percentage of PCNA-positive cells (n = 4 wells per group). (F) Relative mRNA expression of *LGR5*, *ASCL2*, *OLFM4* in jejunal organoids. (G) Relative mRNA expression of Wnt/β-catenin signaling related genes *WNT3*, *AXIN2* and *CTNNB1* in organoids (n = 4 wells per group). (H) Immunofluorescent imaging of Active-β-catenin stained porcine jejunal organoids. Left: representative images (green: Active-β-catenin, blue: DNA, scale bar = 100 μm). Right: Quantification active-β-catenin mean fluorescence intensity (n = 4 wells per group). Graphs represent mean ± SEM. The one-way ANOVA and multiple comparisons in Fisher’s LSD test were performed while asterisks mean statistically significant difference: **P* ≤ 0.05, ***P* ≤ 0.01, ****P* ≤ 0.001, *P* > 0.05 no significance (ns).


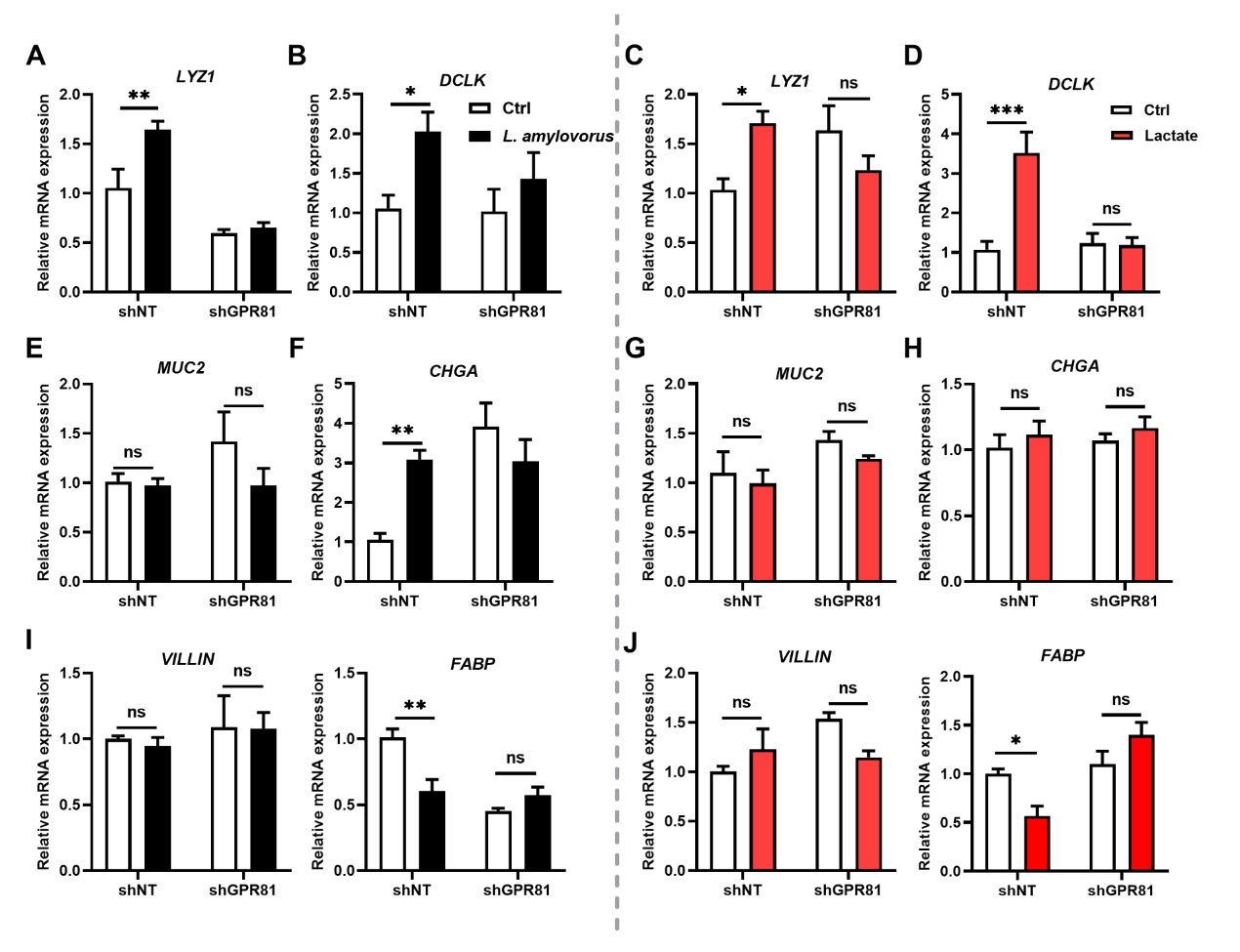


**Figure S10. Effects of *L. amylovorus*-lactate-GPR81 axis on jejunal organoid differentiation.**

(A-H) Effects of 10^6^ CFU of *L. amylovorus* and 5 mM lactate on relative mRNA expression of secretory lineage marker genes including Paneth cell (*LYZ1*), Tuft cell (*DCLK*), goblet cell (*MUC2*) and enteroendocrine cell (*CHGA*) in shNT and shGPR81 group, respectively. (I-J) Effects of 10^6^ CFU of *L. amylovorus* and 5 mM lactate on the relative mRNA expression of absorptive lineages marker genes including enterocyte (*VILLIN* and *FABP*) in shNT and shGPR81 group, respectively. Graphs represent mean ± SEM. The Student's *t*-test was performed between two groups while asterisks mean statistically significant difference: **P* ≤ 0.05, ***P* ≤ 0.01, ****P* ≤ 0.001, *P* > 0.05 no significance (ns).

**Table S1.** Top 5 phyla in the jejunal digesta in Yorkshire and Meishan pigs.

| Top 5 phylum | Relative abundance (%) | | *P* value |
| --- | --- | --- | --- |
|  | Yorkshire | Meishan |  |
| Firmicutes | 75.875 | 95.914 | 0.030 |
| Bacteroidota | 7.166 | 1.517 | 0.792 |
| Proteobacteria | 11.530 | 1.732 | 0.126 |
| Campilobacterota | 3.835 | 0.095 | 0.008 |
| Actinobacteriota | 0.222 | 0.181 | 0.537 |

Note：The data are represented as mean (n = 6). Statistical difference between the 2 groups was calculated by the Mann-Whitney *U* test.

**Table S2.** Top 10 genera in the jejunal digesta in Yorkshire and Meishan pigs.

| Top 10 genus | Relative abundance (%) | | *P* value |
| --- | --- | --- | --- |
|  | Yorkshire | Meishan |  |
| *Lactobacillus* | 14.805 | 45.377 | 0.041 |
| *Clostridium* | 30.743 | 11.069 | 0.132 |
| *Romboutsia* | 12.456 | 2.880 | 0.180 |
| *Escherichia_Shigella* | 1.081 | 0.367 | 0.589 |
| *Sarcina* | 0.752 | 1.567 | 0.818 |
| *Mycoplasma* | 0.320 | 1.822 | 0.699 |
| *Actinobacillus* | 0.189 | 0.146 | 0.485 |
| *Campylobacter* | 2.859 | 0.009 | 0.002 |
| *Turicibacter* | 1.289 | 0.981 | 0.394 |
| *Prevotella* | 0.410 | 0.245 | 0.485 |

Note: The data are represented as mean (n = 6). Statistical difference between the 2 groups was calculated by the Mann-Whitney *U* test.

**Table S3.** The composition and nutrient levels of basal diet（DM basis %）

| **Ingredients** | **Percentage** | **Nutrient levels^b)^** | **Percentage** |
| --- | --- | --- | --- |
| Dry matter | 88.85 | CP | 17.83 |
| Corn | 71.21 | EE | 3.93 |
| Soybean meal | 16.65 | Ca | 0.49 |
| Wheat bran | 8.21 | TP | 0.45 |
| Wheat middlings | 1.00 | AP | 0.22 |
| Premix^a)^ | 1.00 | Lysine | 1.10 |
| Limestone | 0.80 | Methionine + cystine | 0.55 |
| Lysine | 0.61 | ME, MJ•kg-1 | 12.38 |
| CaHPO_4_ | 0.50 | CF | 3.45 |
| Methionine | 0.02 | NDF | 9.58 |
| Soybean oil | 0.00 | ADF | 3.05 |
| Total | 100 | Hemicellulose | 6.53 |
|  |  | IDF | 15.94 |
|  |  | SDF | 0.65 |
|  |  | TDF | 16.59 |

Note: ^a)^ The premix provided the following per kg of diets: vitamin A, 10,800 IU; vitamin D3, 4,000 IU; vitamin E, 40 IU; vitamin K3, 4 mg; vitamin B1, 6 mg; vitamin B2, 12 mg; vitamin B6, 6 mg; vitamin B12, 0.05 mg; biotin, 0.2 mg; folic acid, 2 mg; niacin, 50 mg; D-calcium pantothenate, 25 mg; Fe, 100 mg as ferrous sulfate; Cu, 150 mg as copper sulfate; Mn, 40 mg as manganese oxide; Zn, 100 mg as zinc oxide; I, 0.5 mg as potassium iodide; and Se, 0.3 mg as sodium selenite. ^b)^ In the nutrition level, Calcium, Phosphorus, Lysine, Methionine + cystine and metabolic energy of pigs are calculated values, while other indicators are measured values.

**Table S4. The primers sequence of pig genes used in this study**

| Target gene | Sequence(5’-3’) | Genbank accession. | Product size |
| --- | --- | --- | --- |
| *ASCL2* | F: AAGCTGAGCAAGGTGGAGAC | NM_001122991.1 | 267bp |
|  | R: CTCACAGCCGCTGTCGTC |  |  |
| *AXIN2* | F: GAGGGAGAAATGCGTGGATA | XM_021066739.1 | 152bp |
|  | R: GGTTTCAGCTGCTTGGAGAC |  |  |
| *BMI1* | F: CCTGGAGAAGGAATGGGCCACTTC | XM_021064000.1 | 213bp |
|  | R: GAGGATGAGGAGACTGCACTGGAGTGC |  |  |
| *CTNNB1* | F: ACATGGCTATGGAGCCAGAC | XM_021068566.1 | 153bp |
|  | R: CTTGGGTGGTATCCACATCCT |  |  |
| *CHGA* | F: TCTCCATCCTGCGACATCA | NM_001164005.2 | 111bp |
|  | R: GTTCATCCTCATAACTGCTCTGT |  |  |
| *DCLK* | F: GCTGAAACTGGGCGACTTTG | XM_021065214.1 | 202bp |
|  | R: CTCCTGATCATCACCGCTCC |  |  |
| *GAPDH* | F: GGTCGGAGTGAACGGATTT- | NM_001357943.2 | 133bp |
|  | R: CATTTGATGTTGGCGGGAT |  |  |
| *FABP* | F:ATACCAAGTACAGAGCCAGGAAAACTTTGAGGC | NM_001004046.2 | 213bp |
|  | R: TCCCCAGTCATGGTCTCCATCTCACAC |  |  |
| *LGR5* | F: GCTGGCTGCCGTGGATGC | XM_021090898.1 | 236bp |
|  | R: AGCAGGCGCAGAGGACAAG |  |  |
| *LYZ1* | F: AACTGCTTTGGGTGTCTTGC | NM_214392.2 | 214bp |
|  | R: GGTCTATGATCGGTGCGAGT |  |  |
| *MUC2* | F: CCTTGCTCTCGTGTGGAACA | XM_021082584.1 | 191bp |
|  | R: ACTTCTCCTCGGGCTTGTTG |  |  |
| *OLFM4* | F: CACCAGGAACATTGCCAGAG | XM_003482903.4 | 114bp |
|  | R: TCTTGCCAGCCAACATTAGC |  |  |
| *PCNA* | F: TACGCTAAGGGCAGAAGATAATG | NM_001291925.1 | 192bp |
|  | R: CTGAGATCTCGGCATATACGTG |  |  |
| *VILLIN* | F: TATTATTGGTGTTCGTGCTA | XM_001925167.6 | 103bp |
|  | R: TCTGGAGGAATAGGATACTAA |  |  |
| *WNT2B* | F: TCACTCGTGCCTGTAGCCA | XM_013988158.2 | 217bp |
|  | R: CGACCACAGCGGTTGTTATG |  |  |
| *WNT3* | F: CGTGATTGACAGGCTGAACT | XM_013980865.2 | 173bp |
|  | R: CCACTTGAGATTGGAAATGACT |  |  |

**Table S5. The primer sequence of bacteria used in this study**

| Target gene | Sequence(5’-3’) | References | Product size |
| --- | --- | --- | --- |
| Total bacteria | F: GTGSTGCAYGGYYGTCGTCA | [1] | 123bp |
|  | R: ACGTCRTCCMCNCCTTCCTC |  |  |
| *Lactobacillus* | F: AGCAGTAGGGAATCTTCCA | [2] | 161bp |
|  | R: ATTCCACCGCTACACATG |  |  |
| *L. amylovorus* | F: CAAGCACGATTGGCAAGATG | [3] | 126bp |
|  | R: ATTGGATTCCGCTTCTGTGG |  |  |
| *L. salivarius* | F: TACACCGAATGCTTGCAT TCA | [3] | 138bp |
|  | R: AGGATCATGCGATCCTTAGAG A |  |  |
| *L. mucosae* | F: ACGGACTTGACGTTGGTTTAC | [3] | 156bp |
|  | R: GTGATAGCCGAAACCACCTT |  |  |
| *L. reuteri* | F: GATTGACGATGGATCACCAGT | [3] | 161bp |
|  | R: CATCCCAGAGTGATAGCCAA |  |  |
| *L. delbrueckii* | F: CATGTGCAGACATGCTATCCT T | [3] | 192bp |
|  | R: CTCTGAAGTGCCATGTCTCAGT |  |  |

**Reference**

[1] H. Maeda, C. Fujimoto, Y. Haruki, T. Maeda, S. Kokeguchi, M. Petelin, H. Arai, I. Tanimoto, F. Nishimura, S. Takashiba, *FEMS Immunol. Med. Microbiol.* **2003**, *39*, 81.

[2] E. Khafipour, S. Li, J. C. Plaizier, D. O. Krause, *Appl. Environ. Microbiol.* **2009**, *75*, 7115.

[3] E. Kim, S. M. Yang, B. Lim, S. H. Park, B. Rackerby, H. Y. Kim, *BMC Microbiol.* **2020**, *20*, 96
